# A multi-omic analysis of human naïve CD4+ T cells

**DOI:** 10.1186/s12918-015-0225-4

**Published:** 2015-11-06

**Authors:** Christopher J. Mitchell, Derese Getnet, Min-Sik Kim, Srikanth S. Manda, Praveen Kumar, Tai-Chung Huang, Sneha M. Pinto, Raja Sekhar Nirujogi, Mio Iwasaki, Patrick G. Shaw, Xinyan Wu, Jun Zhong, Raghothama Chaerkady, Arivusudar Marimuthu, Babylakshmi Muthusamy, Nandini A. Sahasrabuddhe, Rajesh Raju, Caitlyn Bowman, Ludmila Danilova, Jevon Cutler, Dhanashree S. Kelkar, Charles G. Drake, T. S. Keshava Prasad, Luigi Marchionni, Peter N. Murakami, Alan F. Scott, Leming Shi, Jean Thierry-Mieg, Danielle Thierry-Mieg, Rafael Irizarry, Leslie Cope, Yasushi Ishihama, Charles Wang, Harsha Gowda, Akhilesh Pandey

**Affiliations:** McKusick-Nathans Institute of Genetic Medicine, Johns Hopkins University School of Medicine, Baltimore, MD USA; Institute of Bioinformatics, International Tech Park, Whitefield, Bangalore India; Department of Molecular & Cellular BioAnalysis, Kyoto University, Kyoto, Japan; Department of Biostatistics, Bloomberg School of Public Health, Johns Hopkins University, Baltimore, MD USA; Sidney Kimmel Comprehensive Cancer Center, Johns Hopkins University School of Medicine, Baltimore, MD USA; National Center for Toxicological Research, Food and Drug Administration, Jefferson, AR USA; National Center for Biotechnology Information, National Institutes of Health, Bethesda, MD USA; Department of Biostatistics and Computational Biology, Dana Farber Cancer Institute, Boston, MA USA; Center for Genomics and Division of Microbiology & Molecular Genetics, Loma Linda University, Loma Linda, CA USA; Department of Biological Chemistry, Johns Hopkins University School of Medicine, Baltimore, MD USA; Department of Pathology and Oncology, Johns Hopkins University School of Medicine, Baltimore, MD USA

**Keywords:** Whole genome sequencing, Epigenomics, Transcriptomics, Proteomics, Phosphoproteomics, Integrative -omics, Innate immunity

## Abstract

**Background:**

Cellular function and diversity are orchestrated by complex interactions of fundamental biomolecules including DNA, RNA and proteins. Technological advances in genomics, epigenomics, transcriptomics and proteomics have enabled massively parallel and unbiased measurements. Such high-throughput technologies have been extensively used to carry out broad, unbiased studies, particularly in the context of human diseases. Nevertheless, a unified analysis of the genome, epigenome, transcriptome and proteome of a single human cell type to obtain a coherent view of the complex interplay between various biomolecules has not yet been undertaken. Here, we report the first multi-omic analysis of human primary naïve CD4+ T cells isolated from a single individual.

**Results:**

Integrating multi-omics datasets allowed us to investigate genome-wide methylation and its effect on mRNA/protein expression patterns, extent of RNA editing under normal physiological conditions and allele specific expression in naïve CD4+ T cells. In addition, we carried out a multi-omic comparative analysis of naïve with primary resting memory CD4+ T cells to identify molecular changes underlying T cell differentiation. This analysis provided mechanistic insights into how several molecules involved in T cell receptor signaling are regulated at the DNA, RNA and protein levels. Phosphoproteomics revealed downstream signaling events that regulate these two cellular states. Availability of multi-omics data from an identical genetic background also allowed us to employ novel proteogenomics approaches to identify individual-specific variants and putative novel protein coding regions in the human genome.

**Conclusions:**

We utilized multiple high-throughput technologies to derive a comprehensive profile of two primary human cell types, naïve CD4+ T cells and memory CD4+ T cells, from a single donor. Through vertical as well as horizontal integration of whole genome sequencing, methylation arrays, RNA-Seq, miRNA-Seq, proteomics, and phosphoproteomics, we derived an integrated and comparative map of these two closely related immune cells and identified potential molecular effectors of immune cell differentiation following antigen encounter.

**Electronic supplementary material:**

The online version of this article (doi:10.1186/s12918-015-0225-4) contains supplementary material, which is available to authorized users.

## Background

The advent of next generation sequencing technologies has enabled broad unbiased surveys of genomic, epigenomic and transcriptomic landscapes in unprecedented detail. The last several years have witnessed tremendous application of these novel high-throughput genomic methods in advancing biomedical sciences. Similarly, advances in mass spectrometry-based approaches have revolutionized biological research by allowing systems level analysis of proteins and post-translational modifications [1]. A recent study by Chen et al. was a first attempt to integrate these high-throughput methods into an integrative personal omics profile of a single individual [2]. This study combined genomic, transcriptomic, proteomic, metabolomic and autoantibody profiles and demonstrated the ability to predict medical risks. However, the data was generated on peripheral blood mononuclear cells, which is a mixed population of different cell types including lymphocytes, monocytes and macrophages. This cellular heterogeneity makes it difficult to gain mechanistic insights into how genome and epigenome level alterations regulate RNA and protein expression. In addition, the ability to interrogate post-transcriptional and post-translational modifications on these biomolecules and correlate changes to their genomic and epigenomic states is complicated by the heterogeneity of cell types.

**Fig. 1:**
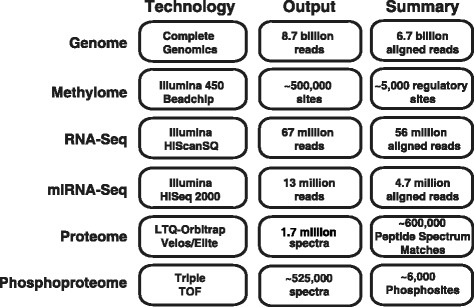
Summary of large-scale omics datasets acquired and the corresponding technologies that were used in this study

In order to overcome these limitations, we carried out a multi-omic study on a purified population of naïve CD4+ T cells from a single individual. Naïve CD4+ T cells are a developmentally synchronized and relatively homogeneous cell population [3–6]. Here we describe the generation of a comprehensive landscape of genome, epigenome, transcriptome and proteome of naïve CD4+ T cells (Fig. 1, Additional file 1: Figure S1). This dataset allowed us to correlate mRNA, miRNA and protein expression patterns of naïve CD4+ T cells with their genomic and epigenomic states. Integration of this multi-omics data also allowed us to interrogate post-transcriptional modifications such as RNA editing in a more precise and detailed manner as well as to identify protein-coding regions that are absent in public databases.

We also carried out a multi-omic comparison of naïve CD4+ T cells and antigen experienced resting memory T cells, which provided mechanistic insights into the epigenetic, transcriptomic and proteomic events that are characteristic of two closely related T cell states. Additional phosphoproteomic analysis also allowed us to determine underlying signaling mechanisms that operate in these two cellular states of T cells.

## Results and discussion

To guide our analysis of naïve CD4+ T cells, we took the following approach. First, we began by characterizing individual omes. This was followed by various pairwise intersections of our data to quantitatively and qualitatively assess relationships between the different data types. This multi-omic integration also allowed us to gain insights into RNA editing, allele-specific expression and to customize our proteomic search strategies. Lastly, we performed a comparative analysis of the methylome, transcriptome, proteome, and phosphoproteome of naïve and memory CD4+ T cells to explore the cellular mechanisms underlying the T cell immunological response. This entailed both a pairwise approach as well as an integrative, pathway level analysis to identify molecular changes following antigen experience of naïve CD4+ T cells.

### Genomic variations and their potential impact on protein coding regions

Our analysis revealed ~3.3 × 10^6^ SNPs, 1.75 × 10^5^ insertions and 1.9 × 10^5^ deletions across the genome (Additional file 2: Figure S2A). Nearly 16,000 SNPs, 130 insertions and 109 deletions were found within protein-coding genes. These sequence alterations led to generation of 107 premature stop codons, 14 nonstop mutations, and 109 frameshifts. Most SNPs within protein-coding regions were synonymous or introduced conservative amino acid changes (Additional file 2: Figure S2B). There were 7,271 non-synonymous changes that predominantly affected protein families associated with extracellular environment including G-protein coupled receptors, immunoglobulins, cell surface/membrane proteins and ion channels (Additional file 2: Figure S2C). This is in agreement with recent observations that potential loss-of-function mutations are quite common in healthy individuals, particularly in gene families that have several paralogs [7]. There are also anecdotal observations that variants in surface or secreted molecules may account for the vast heterogeneity of immune responses and nutrient absorption present in a given population [8–10].

Among genes with non-synonymous changes, *MAP2K3* contained a homozygous variant that introduced a premature stop codon resulting in truncation of most of the *MAP2K3* kinase domain. This is particularly interesting considering that *MAP2K3* is involved in the activation of *p38-MAPK*, which in turn has been shown to promote transcription of multiple genes involved in cell cycle regulation and apoptosis such as *p53* and *MYC* [11, 12]. Another homozygous variant leading to a potential loss of protein function was an insertion within phospholipase *PLCD3* that introduced a frameshift. This mutation resulted in the loss of PLC and C2 domains, which are responsible for hydrolysis of phosphatidylinositol 4, 5-bisphosphate to diacylglycerol and inositol 1,4,5-trisphosphate (IP3). These findings are surprising given that the cells were obtained from a healthy voluntary donor and likely reflect that the affected pathways may have compensatory mechanisms. It is important to note that these two loss-of-function mutations have been recently reported to be frequent in the genomes of healthy individuals from multiple populations [7].

### Transcriptome landscape of naïve CD4+ T cells

**Fig. 2:**
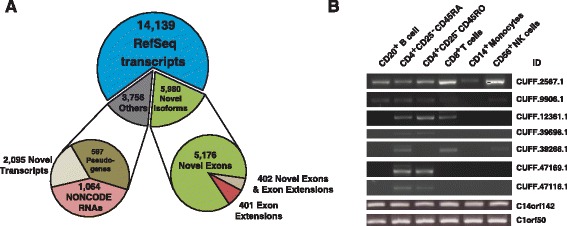
Transcriptome of naïve CD4+ T cells. **a** Pie chart representing the number of diverse classes of transcripts identified in naïve CD4+ T cells. **b** Agarose gel depicting PCR amplified products from RNA isolated from various hematopoietic cell types. Labels at the top show surface markers based on which the cells were purified and labels on the side show cufflinks identifier for each transcript

We sequenced the transcriptome of naïve CD4+ T cells using paired-end RNA sequencing. The abundance of assembled transcripts was estimated using FPKM (Fragments Per Kilobase of exon per Million fragments mapped) and showed a bimodal distribution (Additional file 3: Figure S3). A Gaussian mixture model was applied to model these two distributions. Analysis of transcripts under each peak revealed that the low FPKM peak contained transcripts with few supporting reads that we considered noise. With an FPKM cutoff of two standard deviations from the mean of the left peak (0.860), we found >13,000 transcribed genes represented by ~24,000 transcripts (Fig. 2a; Additional file 4: Table S1). As expected, we detected expression of several cytokine receptors associated with well-defined effector helper CD4+ T cell populations such as Th1 (IL2RA, IL2RB, IL2RG, IFNGR1, and IL12RB1), Th2 (IL4R and IL10RB), and Th17 (IL17RA, IL17RC, IL21R). In general, cytokines, cytokine receptors, major histocompatibility complex, and genes encoding cell surface proteins (e.g., CD4) were expressed at above average levels. As expected, the most abundantly expressed genes included those that code for ribosomal proteins and ribosomal RNA. We identified an additional >2,000 novel transcripts and >6000 novel spliced isoforms absent in our reference annotation (reference annotation composition provided in methods) (Additional file 5: Table S2). We validated the expression of a set of randomly chosen novel transcripts by RT-PCR amplification and sequencing in a panel of primary immune cells including naïve CD4+ T cells. Two of the seven transcripts showed ubiquitous expression across all the tested primary immune cells while others were relatively specific to T cells (Fig. 2b).

We next examined the miRNA profile of naïve CD4+ T cells. miRNA-Seq generated 7.6 million usable reads that were aligned using miRdeep2, identifying 629 known miRNAs as well as 15 putative novel miRNAs (Additional file 6: Table S3). These putative novel miRNAs were identified based on the presence of characteristic features of miRNAs including hairpin formation, sustainable stem-loop structure as well as mature and star sequences as shown in Additional file 7: Figure S4. Over 99.9 % of the reads correspond to 283 miRNAs, suggesting that this forms the primary pool of miRNAs expressed in naïve CD4+ T cells. Using a previously published method, we established that an FPKM of ~73 corresponded to a single miRNA copy per cell, which equates to ~50 % of miRNAs identified being present in at least one copy per cell and ~13 % with at least 100 copies [13].

### Proteomic profile of naïve CD4+ T cells

**Fig. 3:**
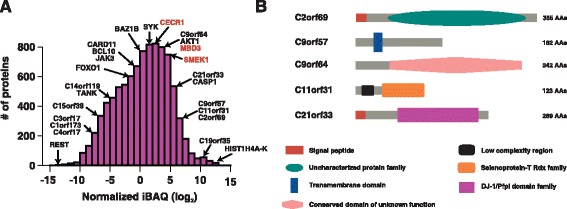
Distribution of proteins expressed in naïve CD4+ T cells based on their relative abundance. **a** Distribution of proteins identified in naïve CD4+ T cells and their corresponding abundance based on normalized iBAQ (log2) values. Genes shown in red indicate those that have been removed in NCBI RefSeq 62, but were detected in our proteomics study. **b** Predicted domain architecture of a subset of RefSeq annotated hypothetical proteins identified in our study

We carried out an in-depth proteomic analysis of naïve CD4+ T cells using multi-dimensional separation methods as well as subcellular fractionation of the nucleus, membrane, and cytosol [1, 13]. In total, proteins encoded by 7,449 genes were identified (Additional file 8: Table S4). To determine the abundance of proteins expressed in naïve CD4+ T cells, we employed intensity-based absolute quantitation (iBAQ) [14] (Fig. 3a). In agreement with transcriptome data, cell surface proteins and constituents of the histocompatibility complex were found to be abundantly expressed. As expected, we identified several cytokine receptors, including IL2RG (CD132), the common gamma chain shared by multiple cytokines receptors (IL2, IL4, IL9, IL15, and IL21) that plays a vital role in T cell signaling [15]. Interestingly, we detected CRLF2, which partners with IL7R and serves as a receptor for TSLP, a major cytokine released by epithelial cells [16–18]. The current paradigm of TSLP signaling states that TSLP influences CD4 T cell function, homeostasis, and pathogenic effects through modulation of dendritic cells. However, given that TSLP is one of the earliest cytokines secreted during inflammation, and CRLF2 and IL7R are co-expressed in naïve CD4+ T cells, these results suggest a likely scenario in which naïve CD4+ T cells could directly sense inflammation locally via the TSLP signaling complex.

We identified 162 proteins annotated as open reading frames (ORFs) in public databases (Additional file 9: Table S5). Some of these proteins were abundantly expressed in naïve CD4+ T cells and have characteristic domain architecture including signal peptides, transmembrane domains and phosphatase domains (Fig. 3b). Future studies may establish some of these molecules as additional novel CD antigens, cytokines and chemokines. Interestingly, we discovered three proteins that have been removed from RefSeq’s protein database (version 71): NP_003917.1, NP_059120.2, and NP_115949.3 (corresponding gene symbols shown in red in Fig. 3a). These proteins were removed due to insufficient protein evidence, transcript evidence, or both. In all three cases, we found mRNA as well as protein evidence supporting their transcription and translation. In the future, such false negatives (i.e., annotations that are suppressed for lack of evidence) can be avoided by integrating transcriptomic and proteomic evidence from published studies.

### Integration of multi-omics datasets

Vertical integration of genome-wide multi-dimensional datasets from an identical background provided us the opportunity to explore 1) how genome sequence and associated epigenetic modifications such as methylation regulate RNA and protein expression in cells; 2) the extent of post-transcriptional modifications such as RNA editing in cells under normal physiological conditions; 3) genes that display allele-specific and isoform-specific expression patterns; and, 4) miRNA expression patterns and their potential influence on the expression of protein targets. Because we gathered data spanning from the genome to the proteome, we could also explore the possibility of novel protein coding regions in the human genome.

### Allele specific expression pattern and RNA editing

By combining whole genome and transcriptome sequencing data from a purified single cell type, we were able to investigate allele-specific expression and RNA editing events. As sequence variants that distinguish two alleles are necessary to draw inferences on allele specific expression patterns, we surveyed all genes containing heterozygous SNPs in accordance with previous approaches [19, 20]. The abundance levels of these genes revealed 84 genes in which only one allele showed detectable expression levels. For genes where both alleles were detected, we defined allele-specific expression as cases where at least 10 reads were mapped and there was at least a 5 fold difference in reads mapped between alleles. Using these criteria, we identified 103 genes where one allele was predominantly expressed. This included known imprinted genes such as *ATP10A* and *SNRPN* along with many additional protein-coding genes, pseudogenes and non-coding RNAs that have not been previously reported as imprinted (Additional file 10: Table S6).

**Fig. 4:**
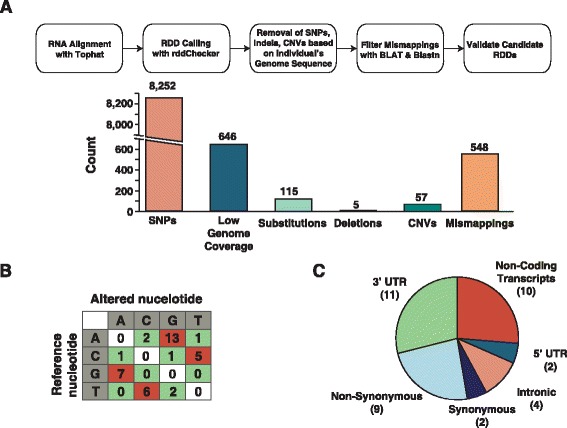
Workflow and summary of RNA editing events observed in naïve CD4+ T cells. **a** The workflow that was used to identify putative sites of RNA editing in naïve CD4+ T cell transcriptome. The number of putative RNA editing sites is shown on the y-axis and reasons for disqualifying them are represented on the x-axis. **b** Matrix showing all the nucleotide substitutions observed in the confirmed RNA editing sites. The numbers highlighted in red indicate canonical changes (A-to-G and C-to-T). G-to-A and T-to-C are also highlighted as those changes are likely canonical edits with incorrect strand assignment of RNA-Seq data. Numbers highlighted in *green* are non-canonical events. **c** Distribution of RNA editing sites based on their location within transcripts

RNA editing is a post-transcriptional mechanism by which the enzymatic deamidation of RNA nucleotides results in altered codon-anticodon pairing. In next generation sequencing, these changes are ultimately reported as adenosine to guanine or cytosine to thymine transitions. These changes can impact biological processes ranging from miRNA binding to protein function. To date, RNA editing has not been studied in a single, purified *primary* cell type, although it has been evaluated in the context of immortalized cell lines and in tissues [21–23]. To identify sites of RNA editing in naïve CD4+ T cells, we developed a pipeline, depicted in Fig. 4a (a more detailed workflow is shown in Additional file 11: Figure S5). Analysis of aligned RNA-Seq data revealed 9,877 potential sites of RNA editing (referred to as RNA DNA Differences, or RDDs) of which 93 % corresponded to a SNP found in whole genome sequencing. RDD containing sequences were then aligned using BLAST or BLAT, which identified another 3 % of RDDs as alignment errors, often involving pseudogenes. Removal of these false positives left 333 potential RNA editing sites. To validate these sites, targeted enrichment and re-sequencing of DNA and RNA was performed on 94 sites. 22 sites were found by DNA re-sequencing to be SNPs not called by our initial WGS. Of the remaining 72 sites validated, 38 were rediscovered as RDDs. For the remaining 34 sites not rediscovered, the edited base was not found at appreciable levels in our targeted sequencing data (all 34 had either 0, 1, or 2 reads supporting the putative RDD), which implies the initial edit may have been a sequencing error. Sites that were validated were predominantly comprised of known, canonical editing sites (A-to-G and C-to-T) (Fig. 4b). To elucidate the functional impact of validated sites, we determined if they were found in protein-coding regions or non-coding regions (i.e., regions considered to be non-coding or untranslated regions of protein-coding regions). As shown in Fig. 4c, the majority of edits occurred in non-coding regions of transcripts with little obvious impact on protein coding regions, which is consistent with a recent study [24]. Interestingly, when examining miRNA binding, we found that 28 miRNA seed sequences were modified by RNA editing and 25 novel sites were created by edits, which may indicate a possible role for RNA editing in miRNA mediated regulation of protein expression.

### Proteogenomic analysis by integrating multi-omics data

**Fig. 5:**
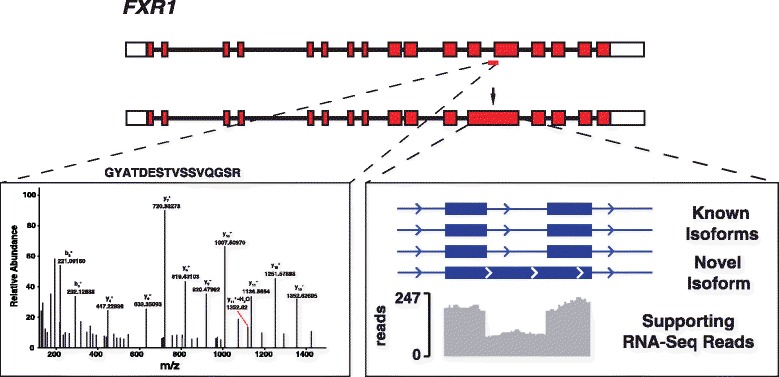
Proteogenomics approach to identify novel protein coding regions. A peptide identified by proteogenomics that mapped to an intronic region of *FXR1* gene. MS/MS spectra of the peptide and read density from RNA-Seq data that supports the new isoform are depicted

Availability of high coverage genomic, transcriptomic and proteomic data allowed us to annotate novel protein features as well as refine existing annotations [25]. Using our proteogenomics pipeline outlined in Additional file 12: Figure S6, we searched unmatched tandem mass spectra against custom peptide/protein sequence databases that incorporated genomic variants revealed by whole genome sequencing. In addition, we built custom protein databases comprised of a 6-frame translation of human genome build 19 and a 3-frame translation of our RNA-Seq reference annotation (comprised of mRNAs, ncRNAs, lncRNAs, and pseudogenes). In our proteome dataset, we identified >10,000 peptides that spanned known exon splice junctions and identified 17 peptides providing evidence for the coding potential of 11 novel exons, all of which were identified from RNA-Seq data as well. An example of a novel isoform of FXR1 identified using RNA-Seq as well as proteomic data is shown in Fig. 5. We also identified 2 potential upstream ORFs based on 2 peptides identified in untranslated regions of known mRNAs (Additional file 13: Table S7). To characterize and discover new translational start sites, we searched for N-terminal acetylation of proteins. We identified 1,740 N-terminal acetylated peptides, which confirm existing annotations of translation start sites for 1,720 gene products. To locate alternative start sites, a database containing alternate methionine residues immediately upstream or downstream of known translational start sites was created. From this, 20 alternate N-termini acetylated peptides downstream of currently annotated protein sequences were identified, providing experimental support for previously unknown translation start sites.

Signal peptides are an integral part of post-translational translocation of proteins to specific locations within or outside the cell. Presently, database search-driven algorithms are unable to successfully predict cleavage sites and identify signal peptides due to difficulties in identifying unexpected trypsin-cleaved amino acid signal sequences [26]. To overcome this difficulty, we incorporated tryptic peptides preceding the cleavage sites based on predicted signal sequences from SMART [27], SignalP4.0 [28], and cleavage sites annotated in HPRD [29] into our analysis pipeline. We identified 35 known and 24 novel signal peptide cleavage sites (Additional file 14: Table S8). Our search against a translated pseudogene database identified 3 peptides uniquely mapping to 3 pseudogenes (Additional file 15: Table S9) suggesting that they are actually putative novel protein coding genes. These findings illustrate that a high coverage of the cellular proteome with high resolution mass spectrometry-derived data allows for a systematic exploration of the information flow from genome to proteome that can augment genome annotation.

To explore the relationship between protein and transcript abundance in naïve CD4+ T cells, we correlated the iBAQ values of proteins and transcript FPKM values. A Spearman’s correlation of 0.35 between FPKM and iBAQ values was found, indicating poor correlation between transcript and protein abundance (Additional file 16: Figure S7). To investigate the role of miRNAs in transcriptional or translational repression of genes expressed in naïve CD4+ T cells, miRNA levels were quantified and compared to iBAQ and FPKM values. From a global perspective, when considering the protein and transcript abundance of genes that are potential targets of miRNAs, we found that there was a steady decrease in the average protein level of targeted genes with increasing miRNA read counts (Additional file 17: Figure S8A). However, at the transcript level, this trend was not as apparent (Additional file 17: Figure S8B).

### DNA methylation patterns and their influence on RNA and protein expression

Methylation of DNA is one of the most well-known regulatory mechanisms of gene expression, with increased methylation often corresponding to diminished transcriptional activity. Here, we assayed genome-wide methylation patterns using an Illumina 450 K BeadChip array. The global methylation profile exhibited a bimodal distribution with 39 % of the assayed sites showing hypomethylation (beta value < 0.33) and 52 % showing hypermethylation (beta value > 0.66). In promoter regions (defined as 0–1500 bases upstream of a transcription start site (TSS)), 69 % of the sites were hypomethylated and 23 % were hypermethylated. In general, the methylation levels were lower near the TSS and rose through the gene body and 3′ UTR (Additional file 18: Figure S9).

Methylation levels were also compared with nearby transcript and protein levels (using FPKM and iBAQ as measurements for transcripts and proteins, respectively). This revealed that methylation of promoter regions showed a weak correlation with lower transcriptional levels of corresponding genes (Additional file 19: Figures S10A and S10B). No correlation was observed between protein abundance and promoter methylation (Additional file 19: Figure S10C) or methylation of various gene regions (Additional file 19: Figure S10D). We hypothesize the lack of correlation between methylation and protein levels found here is due to our global analysis. Because not all genes are regulated by methylation, and there is a low correlation between transcript and protein levels, we believe the true signal of protein levels trending with methylation levels is obscured. To rectify this, we feel the correct stratification of data combined with additional experimental work is needed to detect changes in protein abundance as a function of methylation.

### A multi-omic comparison of naïve CD4+ T cells with antigen-experienced resting memory CD4+ T cells

#### Epigenetic regulation of T cell differentiation

Naïve CD4+ T cells differentiate into several lineages of CD4+ helper T cells after experiencing an antigen - one lineage being resting memory CD4+ T cells. Resting memory CD4+ T cells embody the molecular changes induced by antigen exposure of naïve CD4+ T cells. To dissect the underlying molecular mechanisms that regulate this transition, we carried out a multi-omic comparative analysis of these two closely related cell populations. To identify promoter methylation dependent regulatory mechanisms, we intersected methylome and transcriptome data from both cell types and found genes that show differential methylation patterns that may influence gene expression. Promoter regions of the kinases LYN and SGK1 as well as the methyl-transferase/hydrolases METTL7A and DDAH2 were hypomethylated with concurrent increases in gene expression levels in naïve CD4+ T cells relative to memory T cells (Additional file 20: Figure S11). Conversely, promoter regions of CCR6 and chemokine CCL5, which is essential for migration of CD4+ T cells, were hypermethylated. Coupled with the decreased expression levels of these genes in naïve CD4+ T cells, these results suggest that there are specific molecular changes in methylation and gene expression involved in the transition from naïve CD4+ T cells to resting memory T cells that is consistent with previous observations [30–32].

We next explored the role of non-promoter associated methylated sites in our dataset. Potential regulatory DNA methylation sites were identified by comparing DNA methylation and gene expression patterns in hundreds of tumors from TCGA datasets and selecting sites with an inverse methylation and gene expression correlation (TCGA, https://tcga-data.nci.nih.gov/tcga/) [33, 34]. For each regulatory site identified from the TCGA dataset, we examined differences in DNA methylation and gene expression between naïve CD4+ T cells and memory T cells (Additional file 21: Table S10). This approach allowed us to enrich for DNA methylation sites, genes and pathways associated with CD4+ T cell biology (Additional file 22: Table S11). For example, within the TCR pathway, many genes known to be critical for T cell activation were highly methylated at regulatory sites with concurrent decreases in expression levels in naïve cells as compared to memory cells. This suggests an epigenetic regulation of this critical pathway required for T cell differentiation.

### Proteomic and phosphoproteomic differences between naïve and memory T cells

**Fig. 6:**
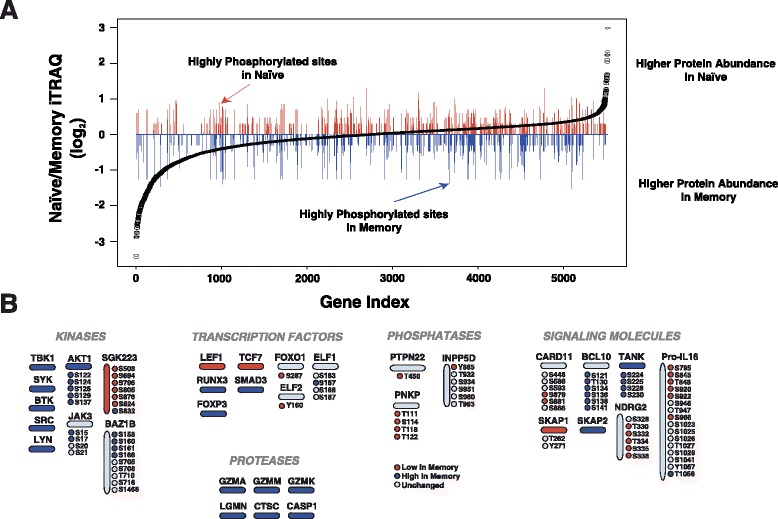
Differential methylation, gene/protein expression and phosphorylation pattern between naïve CD4+ T cells and memory T cells. **a** Differential protein expression and phosphorylation pattern between naïve and memory CD4+ T cells. The *black data points* represent relative protein expression levels between naïve and memory T cells while the red and blue bars indicate relative phosphorylation levels in naïve and memory T cells, respectively. **b** Examples of kinases, phosphatases, proteases, transcription factors and signaling molecules that showed significant differences in their expression level and/or phosphorylation levels between naïve and memory T cells. Vertical and horizontal ovals represent proteins and circles represent phosphorylation sites. Higher levels in memory cells are indicated in blue, lower levels are indicated in *red* while unchanged abundance is indicated by *grey color*

To investigate signaling differences between naïve CD4+ T cells and memory CD4+ T cells we employed comparative proteomic and phosphoproteomic approaches. Quantitative proteomics was performed using iTRAQ [35] based methodology, which provides a measure of protein abundance between samples. In addition, we enriched phosphopetides from both cell types using a TiO_2_-based strategy and quantified their differences based on ion abundance [27, 36]. As shown in Fig. 6a, several proteins were found to be differentially expressed between naïve and resting memory T cells. Interestingly, many proteins displayed differential phosphorylation levels between the two cell types without any alteration in protein abundance. Notable examples of differentially phosphorylated proteins include two poorly characterized kinases, SGK223 and BAZ1B, which showed an inverse phosphorylation pattern between the two cell types (Fig. 6b). SGK223 showed elevated protein as well as phosphorylation levels in naïve CD4+ T cells, including two previously unreported sites, Ser508 and Ser805. Memory cells expressed higher protein levels of kinases that are known to play critical roles in T cell activation, including LYN, SYK, BTK and TBK1. Higher expression of these kinases in memory cells is consistent with the notion that antigen exposure of memory cells leads to a potent and faster immune response by virtue of having elevated levels of signaling molecules [38]. Our proteomic analysis also indicated differential phosphorylation of well-studied adaptor proteins in T cell signaling. For instance, caspase recruitment domain-containing protein 11 (CARD11) is a multi-domain scaffolding molecule responsible for mediating NF-kB activation during T cell activation [39, 40]. Phosphorylation of Ser552, Ser564, and Ser657 in CARD11 is known to induce CARD11-mediated NF-kB activation while phosphorylation of Ser649 is known to down-regulate NF-kB activation [41, 42]. Most interesting, however, were proteins that were not differentially expressed between samples, but were differentially phosphorylated. BAZ1B, a recently discovered tyrosine kinase, exhibited similar protein levels in both cell types but showed elevated phosphorylation levels in memory cells. Six differentially phosphorylated sites were identified on BCL10, a key component of the CARD11-BCL10-MALT1 complex that regulates NF-kB activation [43, 44]. The novel sites Ser121, T130, S134, S136, Ser141 and previously known Ser138 were all highly phosphorylated in memory CD4+ T cells. Ser138 negatively regulates NF-kB activation and thus the increased phosphorylation of this site in naive cells is consistent with antigen inexperience where NF-kB activation is limited [45].

During T cell activation, proteolytic cleavage by caspase-3 converts pro-IL16 to IL16, a proinflammatory chemoattractant that stimulates cell migration [46, 47]. The IL16 precursor pro-IL16 is a nuclear and cytoplasmic scaffolding protein involved in cell cycle arrest through association with a broad range of proteins including HDACs and ion channels in resting T cells [48]. In our study, pro-IL16 protein expression levels were comparable between naïve and memory CD4+ T cells. However, phosphorylation patterns were different between these two closely related cells. We have identified 16 phosphorylation sites, 10 of which are novel, in pro-IL16. Interestingly, 6 of the 16 sites were highly phosphorylated in naïve CD4+ T cells while Thr1058 was highly phosphorylated in memory cells. These findings parallel the different roles pro-IL16 plays in naïve and memory CD4+ T cells. Through a combined analysis of quantitative proteomic and phosphoproteomic data, we were able to probe T cell biology and gain novel insights into phosphorylation mediated regulation of these two cell phenotypes. Together, these findings suggest an active role for new kinases and reveal signaling events that may be crucial in T cell activation.

**Fig. 7:**
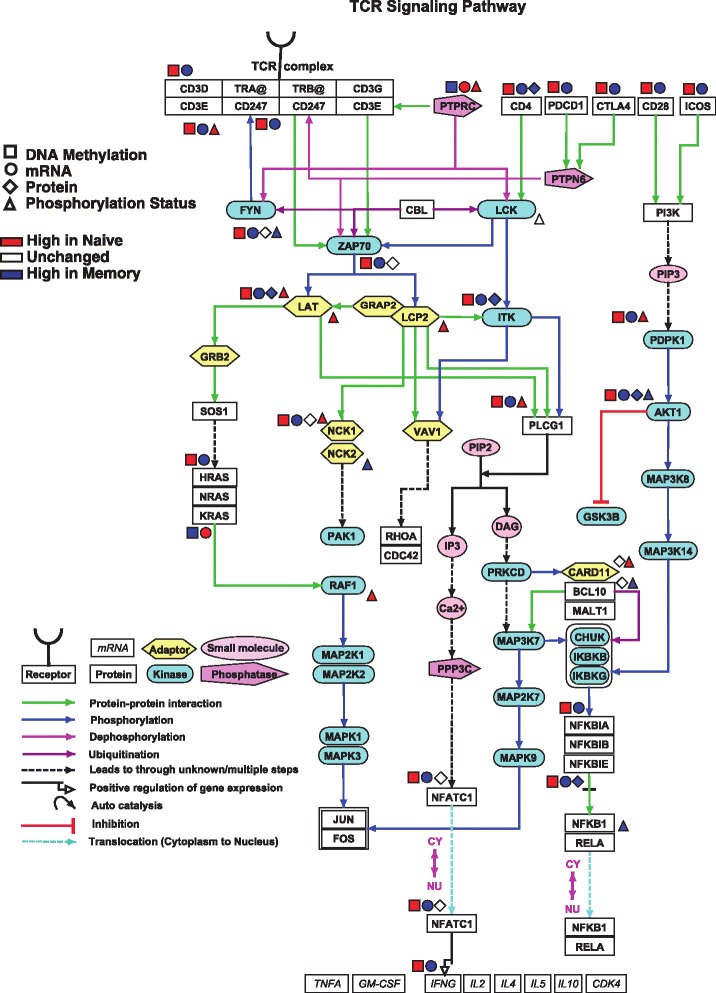
T cell receptor signaling pathway showing downstream mediators regulated by DNA methylation, gene/protein expression and/or protein phosphorylation. T cell receptor signaling pathway depicting genes with their corresponding promoter methylation status, gene and protein expression levels and phosphorylation status between naïve and memory T cells. Promoter methylation status, transcriptomic, proteomic and phosphoproteomic measurements were imported into GeneSpring and overlaid onto the TCR signaling pathway to identify molecular signatures underlying the transition from naïve CD4+ T cells to memory CD4+ T cells. Different *shapes* are used to denote promoter methylation, mRNA, protein and phosphorylation status. *Red* indicates high in naïve CD4+ T cells, *blue* indicates high in memory CD4+ T cells and the unchanged are depicted in *white*. Different *colored arrows* are used to depict various modes of regulation of pathway components

By integrating data from methylome, transcriptome, proteome, and phosphoproteome, we obtained a comprehensive view of molecular events that regulate naïve and memory T cells (Fig. 7). To assess the utility of an integrated pathway, we evaluated several key signaling molecules in the context of immune function. Given that both cell populations are in a resting state, LCK, a key downstream kinase in the TCR pathway, is unchanged both at the protein and phosphorylation levels. However, CD4, ITK, and AKT1 were hypermethylated in naïve CD4+ T cells, with concomitant decreases in transcript and protein levels. Protein tyrosine phosphatase, receptor type, C (PTPRC, also known as CD45) and KRAS were two notable exceptions to this pattern. High protein levels of KRAS have recently been implicated in lowering the TCR-activation threshold in T cells [49]. Here, we found hypomethylation of KRAS in memory cells along with high transcript and protein levels. In contrast, in naïve cells KRAS is hypermethylated and has lower transcriptional levels, consistent with a higher TCR activation threshold. Taken together, observations from this integrated approach are consistent with what is known about the TCR pathway, demonstrating the power of a multi-omics dataset to gain comprehensive molecular insights.

### Integration with other datasets

Lastly, we sought to evaluate our data alongside recently published studies on naïve and memory CD4+ T cells to examine the concordance and to explore how our data could complement available datasets.

To investigate the relationship between methylation and transcription in naïve and memory CD4+ T cells, *Komori* et al. utilized RNA-Seq and targeted bisulfite sequencing of 2,100 genes. They reported 132 genes with differential methylation between naïve and memory CD4+ T cells. Of these 132 genes, 39 exhibited a reduction in methylation with a concurrent increase in transcript abundance in naïve versus memory cells. Unfortunately, quantitative methylation values were available for only 40 of these genes, thus for our analysis we mapped their ‘increased, no-change, and decreased’ methylation designations for the 132 genes into a 1, 0, and −1, respectively, for our analysis. When comparing methylation levels across the two studies, we observed a Pearson correlation of 0.42 (Additional file 23: Figure S12A). This correlation was increased to 0.61 when only the 40 genes where methylation values were available were compared (Additional file 23: Figure S12B).

Next, we evaluated how well methylation levels correlated with expression of transcripts derived from neighboring genes. For this, we limited our comparison to resting memory CD4+ T cells and unactivated naïve CD4+ T cells and selected genes that exhibited an inverse relationship between methylation and transcription. Although *Komori* et al. described an inverse relationship between methylation levels and transcription of these genes; we did not observe a similar general trend in our data (Additional file 23: Figure S12C). To determine if this was due to global differences in transcript levels between each study, we compared the RPKM values from *Komori* et al. with FPKM values from our study. We derived a Pearson correlation coefficient of 0.58 indicating transcript levels between each respective experiment were somewhat consistent (Additional file 23: Figure S12D). Additional experiments will be required to resolve these apparent disparities.

A recent publication by *Graessel* et al. characterized cell surface proteins of naïve and activated naïve CD4+ T cells in which they employed an enrichment strategy targeting N-glycosylated surface proteins to identify 173 cell surface proteins by mass spectrometry. This was complemented with flow cytometry to identify an additional 123 cell surface proteins, for a total of 229 proteins expressed on the surface of naïve and activated naïve CD4+ T cells. Because of the deep proteomic profiling of naïve CD4+ T cells in our study, we sought to complement *Graessel* et al.*’s* surface atlas. Additionally, because we performed a comparative analysis of naïve and resting memory CD4+ T cells, we wanted to determine which changes may be transient during naïve CD4+ T cell activation, and which protein changes persist to the resting memory state.

First, to expand upon *Graessel* et al.*’s* surface atlas, we identified proteins from our study using their bioinformatic strategy of retaining proteins annotated as ‘membrane’ or ‘secreted’ in the UniProtKB/Swiss-Prot databases. This analysis identified 1,767 candidate proteins in our dataset. To make a more conservative estimate, we employed an additional criterion of retaining proteins annotated as membrane or extracellular by GO annotation, resulting in 1,166 proteins. We applied the same filters to *Graessel* et al.*’s* proteomic data, and found 146 proteins meeting these criteria. Between our datasets, there was an overlap of 104 cell surface proteins, with 42 proteins being found only in *Graessel* et al. and 1,062 proteins being exclusive to our data (Additional file 24: Figure S13A). The disparity in depth is likely due to *Graessel* et al. adopting a N-glycoprotein protein enrichment strategy as opposed to the global, deep fractionation methods employed in our study.

Next, we compared the changes measured by *Graessel* et al. following naïve CD4+ T cell activation with αCD3/αCD28. *Graessel* et al. reported a cluster of 108 proteins with increased cell surface expression following 24 h of activation. In our data, we found the protein expression of these 108 proteins to be, on average, increased in resting memory CD4+ T cells (Additional file 24: Figure S13B). Many of these genes are highly associated with human T cell differentiation and functions such as proliferation (e.g., CD2 superfamily molecules, TNSFS14, CD147), energy molecule transport (e.g., GLU3, SLC genes), cellular adhesion and migration (e.g., CD29, CD226), and components of antigen processing and presentation to include CD74 (invariant chain) and HLA molecules.

## Conclusions

Technological advances in the fields of genomics, transcriptomics, and proteomics in the last decade have enabled us to carry out broad, unbiased studies surveying the genome, transcriptome, and proteome. Several studies in the recent past have shown that, sometimes, a single study employing many of these approaches can rediscover most of what was discovered by decades of research in addition to providing large volumes of novel data. This is particularly evident in cancer genome sequencing studies where a single study of a given cancer can reveal most mutations that took years of research efforts by multiple investigators as well as revealing novel gene mutations. With the rapid pace of technologic development, the challenge now is to determine ways to integrate data from these multi-omics approaches. This would revolutionize biomedical research and enable us to explore how a given genotype influences phenotype through various molecular mediators. In addition, this would also allow us to employ systems biology approaches to better understand biological systems in health and disease. Our study is a small step towards encouraging such multi-omics approaches to study biological systems.

Through analysis of the transcriptome, we measured the baseline expression levels of transcripts in primary naive and memory CD4+ T cells in addition to revealing several novel transcripts. Notably, these transcripts were shown to be differentially expressed across several primary immune cell populations through RT-PCR amplification of each respective transcript. Small RNA analysis revealed 629 known miRNAs expressed in naive CD4+ T cells as well as 15 putative novel miRNAs. The proteome was extensively profiled and quantified through various fractionation strategies including sub-cellular fractionation. This established a baseline abundance estimate of over 7,000 genes expressed in naive CD4+ T cells and revealed abundantly expressed proteins with little known function roles. Using a proteogenomic analysis we uncovered 11 new coding exons and 2 translated ORFs in the 5′ UTR of known mRNAs. We also confirmed the translational start site of 1, 720 proteins and discovered a number of previously unknown start sites. Our analysis identified 24 new signal peptide cleavage sites, and provided protein coding evidence for 3 pseudogenes. Integrating RNA-Seq with whole genome sequencing allowed us to explore RNA editing and allele specific expression patterns in primary human cells. We found low levels of canonical RNA editing, with most edits occurring within non-coding regions as well as 103 genes that demonstrate transcription patterns consistent with allele specific expression.

To probe the mechanisms underlying antigen experience of naive CD4+ T cells, comparisons of the epigenome, transcriptome, proteome, and phosphoproteome were carried out against resting memory CD4+ T cells. This revealed methylation sites exhibiting regulatory tendencies within genes involved with an immune response such as LYN, SGK1, CCR6, and CCL5. Comparative proteomics using iTRAQ and phospho-enrichment revealed differential expression and phosphorylation levels of key immune related genes. Furthermore, we found that many proteins with comparable protein levels displayed significantly different phosphorylation patterns between the two cell populations. This indicates the regulatory potential associated with various phosphorylation sites and demonstrates the power of targeted proteomics to profile post-translational modifications.

Lastly, our effort revealed several challenges associated with integrating multi-omic data. The comparison and integration across various omics datasets is easier if the depth of coverage is similar; however, each platform might have biases or issues with sensitivity that preclude a uniform depth. In addition, comparing different omics datasets poses challenges because normalizing outputs from various technological platforms to provide meaningful comparisons is not always possible. Computational algorithms are an essential part of omic data analysis, with each generating its own share of false positives as well as false negatives. Such errors could give rise to artificial conflicts across datasets. However, we foresee that as technologies mature and data analysis pipelines and methodologies become more robust, these challenges will be overcome in the future. For developing such methods, availability of multi-omics datasets from similar or identical biological background is fundamental. This will mitigate bias due to biological variation and allow researchers to address inconsistencies in data acquisition as well as data analysis strategies. In that context, our dataset should prove useful for informatics researchers developing multi-omics data analysis pipelines.

## Methods

Additional details may be found in Additional file 25.

### Cell isolation and sample preparation

Blood samples were obtained for isolation of hematopoietic cells after obtaining informed consent from a healthy subject. Samples were anonymized prior to analysis and data deposition. This study was approved by the Johns Hopkins University’s Institutional Review Board for use of human samples. Peripheral blood was collected in sodium heparin containing vacutainer blood collection tubes and peripheral blood mononuclear cells (PBMCs) were enriched by Ficoll gradient. Naïve CD4+ T cells were isolated using magnetic beads following manufacturer’s instructions (Miltenyi Biotec #130.094.131) and assessed using FACS Calibur, with the average purity of each isolation being ~95 % (Additional file 26: Figure S14A). Resting memory cells were isolated by enriching pan resting CD4+ T cells followed by CD45RO depletion of resting memory CD4+ T cells, with the average purity of each isolation also being >95 % (Additional file 26: Figure S14B). Isolated cells were washed with cold PBS in large volumes and stored in −80 °C. Genomic DNA was isolated from cells using the FlexiGene DNA kit, RNA was isolated from cells using RNeasy mini kit (QIAGEN) and small RNAs were isolated using miRNeasy mini kit (QIAGEN).

### Sequencing and data processing

Whole genome sequencing was performed by Complete Genomics (CG). 35 bp paired-end reads were generated by CG and mapped to human reference genome 19. Total RNA was purified from naïve CD4+ T cells and a RNA-Seq library was constructed using Illumina’s TruSeq kit and sequenced on HiScanSQ. Reads were aligned using Tophat version 2.0.10 and transcripts generated by Cufflinks using a reference annotation comprised of Illumina’s iGenomes, Mark Gerstein’s Pseudogene annotations (version 61), and NONCODE long non coding RNA (lncRNA). For miRNA sequencing, small RNAs were gel excised, amplified, sequenced on a HiSeq2000 as single-end reads. Reads ≥18 bp following adapter trimming were kept, and contaminant sequences were removed prior to alignment against miRBase 20 with miRDeep2.

### DNA methylation analysis

DNA was bisulfite converted and analyzed on Illumina’s HumanMethylation450 BeadChip Array and normalized using the methylumi R package. Differential DNA methylation was quantified by determining the difference of averaged DNA methylation values between probes and grouped into genomic regions. RMA was used for all array gene expression analysis and the difference in expression was computed using average values from replicate runs. DMRs closest to genes and before the nearest TSS were plotted against transcript fold change. To identify CpG sites which are known to regulate gene expression, we used the Spearman correlation coefficient to evaluate the association between DNA methylation and mRNA expression in primary tumors from the Cancer Genome Atlas. From these datasets, we derived an informer set by identifying genes that showed an inverse relationship between promoter methylation and gene expression. We derived DNA methylation and corresponding gene expression differences between naïve and memory CD4+ T cells at CpG sites of this gene subset.

### Nuclear preparation

Cells were washed in cold PBS and centrifuged at 150× g. The cell pellet was resuspended in hypotonic lysis buffer (20 mM HEPES, 10 mM KCl, 2 mM MgCl_2_, 2 mM CaCl_2_, pH7.4, protease inhibitor), ruptured by nitrogen cavitation by pressurizing at 1,500 psi, and crude nuclei were collected by centrifugation. The pellet was washed with nuclear wash buffer (250 mM sucrose in hypotonic buffer) and nuclei collected by centrifuging at 1,000× g for 10 min at 4 °C and stored at −80 °C until further use.

### Protein fractionation, trypsin digestion, and LC MS/MS analysis

Whole cell lysates were resolved by SDS-PAGE, gel bands were cut, and in-gel tryptic digestion was performed overnight. Peptides were extracted from the gel pieces, dried, and analyzed by LC-MS/MS. Protein fractionation was also carried out on GELFREE 8100 fractionation system according to manufacturer’s protocol. Each fraction was then subjected to SDS-PAGE, followed by in-gel tryptic digestion as described above. Strong-cation exchange (SCX) as well as basic reversed-phase liquid chromatography (bRPLC) following in-solution trypsin digestion of protein lysate and LC MS/MS analysis were performed as described previously [50]. For iTRAQ based quantitative proteomics experiments, 75 μg of in-solution digested peptides from both naïve and memory T cells were labelled according to manufacturer’s protocol.

### MS data processing and database searching

MS data were processed using Proteome Discoverer 1.3 (Thermo Fisher Scientific) and searched using Mascot version 2.2 and Sequest search algorithms against a human RefSeq protein sequence database and proteogenomics analysis was conducted as described previously [51, 52]. Identified peptides were subjected to a 1 % false discovery rate (FDR) filter using the decoy database method.

### Phosphopeptide enrichment and label-free quantitation

Lysates from naïve and memory CD4+ T cells were digested using in-solution trypsin digestion and phosphopeptides were enriched using TiO_2_-based enrichment protocol. Phosphopeptides were desalted by Stage-Tip and subjected to LC-MS/MS using Triple-TOF mass spectrometer with a long gradient. Spectra were searched against human protein database using Mascot and ProteinPilot and peptides were identified by setting a threshold of 1 % FDR. Phosphopeptide abundance was quantitated by label-free method using MassNavigator. AreaScore(L) was used to assess the confidence of the area quantitation and areas were normalized based on a total summed intensity of proteomic analysis.

## Data availability

Mass spectrometry derived proteomics data has been deposited to the ProteomeXchange Consortium (http://proteomecentral.proteomexchange.org) via the PRIDE partner repository with the dataset identifier PXD000376. All sequencing data has been deposited to the Short Read Archive under the BioProject identifier PRJNA234019.

## Additional files

Additional file 1: Figure S1. Data generated on naïve CD4+ T cells and memory CD4+ T cells. Basic metrics assaying the quality of generated data are provided for each dataset generated. (PDF 60 kb) Additional file 2: Figure S2. Sequence variants identified in the genome and classification of SNPs in protein coding regions. (A) Total number of SNPs, insertions, deletions and substitutions identified from whole genome sequencing data. (B) Proportion of synonymous and non-synonymous SNPs identified in the coding region of the genome among non-synonymous SNPs (nsSNPs), proportion of conservative and non-conservative homozygous and heterozygous changes are shown. (C) nsSNP frequency across various molecular classes of genes. (PDF 68 kb) Additional file 3: Figure S3. Distribution of assembled transcripts based on abundance (FPKM) and corresponding read density. The x-axis represents abundance threshold (FPKM) and y-axis represents read density. The red and green lines represent the Gaussian Mixture Model that was applied to assembled transcripts. The abundance threshold (represented by vertical dotted line) separates transcripts identified as true positives on the right (supported by more reads) from those identified as potential false positives on the left (supported by less reads). The two screenshots taken from genome browser demonstrates examples of high read density and low read density. (PDF 226 kb) Additional file 4: Table S1. A list of genes expressed in naïve CD4+ T cells. (XLSX 403 kb) Additional file 5: Table S2. A list of novel transcripts and novel spliced isoforms identified in naïve CD4+ T cells. (XLSX 9927 kb) Additional file 6: Table S3. A list of known and novel miRNAs identified in naïve CD4+ T cells. (XLSX 22 kb) Additional file 7: Figure S4. Example of a miRNA identified and the distribution of reads across a predicted hairpin structure. Putative novel miRNA identified from small RNA-Seq. Predicted stem loop structure, mature region and star regions are shown. (PDF 50 kb) Additional file 8: Table S4. A list of proteins identified in naïve CD4+ T cells. (XLSX 634 kb) Additional file 9: Table S5. A list of uncharacterized proteins annotated as open reading frames in public databases that were identified in naïve CD4+ T cells. (XLSX 16 kb) Additional file 10: Table S6. A list of genes displaying allele specific expression. (XLSX 15 kb) Additional file 11: Figure S5. Strategy employed for determining potential RNA editing sites . The workflow that was followed to filter out false positive RNA-editing sites is shown. Examples of false positives due to mismapping of reads and uncalled SNPs is demonstrated with sequence alignment. Abbreviations (SNP: single nucleotide polymorphism; RDD: RNA-DNA difference; CNV: copy number variation). (PDF 65 kb) Additional file 12: Figure S6. Proteogenomic pipeline. Custom protein databases that were used for searching tandem mass spectrometry data for proteogenomic annotation. (PDF 59 kb) Additional file 13: Table S7. A list of peptides supporting novel exons or exon extensions identified using proteogenomics strategy. (XLSX 9 kb) Additional file 14: Table S8. A list of known and novel signal peptide cleavage sites identified for membrane/secreted proteins expressed in naïve CD4+ T cells. (XLSX 11 kb) Additional file 15:Table S9. A list of annotated pseudogenes that encode proteins in naïve CD4+ T cells with the corresponding peptide evidence from proteomics data. (XLSX 9 kb) Additional file 16: Figure S7. Comparison of a protein’s abundance versus its transcriptional abundance. Scatterplot of protein abundance versus transcript abundance is shown. Transcript abundance is represented as FPKM (log2) on the x-axis and protein abundance is represented as normalized iBAQ (log2) on the y-axis. (PDF 487 kb) Additional file 17: Figure S8. Correlation between miRNA read count, transcript, and protein abundance. (A) Protein abundance versus miRNA read count. The x axis for each circle represents all miRNAs identified with a given read count while the y axis corresponds to the average iBAQ value of the genes targeted by those miRNAs with the specified read count. The dashed red line represents the iBAQ value of genes that are not targeted by any miRNAs identified in this study and serves as a background, reference level of protein expression. The black line is a linear regression of iBAQ values versus the miRNA read count. (B) Transcript abundance versus miRNA read count. The x axis for each circle represents all miRNAs identified with a given read count while the y axis corresponds to the average FPKM value of the genes targeted by those miRNAs with the specified read count. The dashed red line represents the FPKM levels of genes that are not targeted by any miRNAs identified in this study and serves as a background, reference level of transcript abundance. The black line is a linear regression of FPKM values versus miRNA read count. There is no obvious correlation between FPKM values and miRNA read counts. (PDF 150 kb) Additional file 18: Figure S9. DNA methylation pattern across various gene features on all protein coding genes in naïve CD4+ T cells. The x-axis represents various gene features in protein coding genes (TSS1500 - 1,500 bp upstream of transcription start site, TSS200 - 200 bp upstream of transcription start site, 5′ UTR – 5′ untranslated region, 1^st^ exon, gene body and 3′ UTR – 3′ untranslated region) and the y-axis represents beta value which is an estimate of methylation level. Box-Whisker plots show median methylation levels at each gene feature across all protein coding genes in naïve CD4+ T cells. (PDF 1313 kb) Additional file 19: Figure S10. Correlation of methylation level at different gene features with transcript and protein expression levels. (A) Promoter methylation level and corresponding transcript abundance Methylation levels are represented by Beta values and transcript abundance is represented by FPKM values. (B) Scatterplots showing methylation levels at different gene features and transcript abundance. Methylation levels are represented on the x-axis and transcript abundance is represented on the y-axis. The red dot represents the mean methylation levels and transcript abundances. Spearman’s correlation coefficent is represented below each scatterplot. (C) Promoter methylation level and corresponding protein abundance (iBAQ values from Fig. 4). (D) Scatterplots showing methylation levels at different gene features and protein abundance. Methylation levels are represented on the x-axis and protein abundance is represented on the y-axis. The red dot represents the mean methylation levels and protein abundances. Spearman’s correlation coefficent is represented below each scatterplot. (PDF 1087 kb) Additional file 20: Figure S11. Correlation of methylation and transcript levels of genes between memory and naïve CD4+ T cells. (A) Intersection of promoter methylation and mRNA expression data from naïve and memory T cells. Genes marked in red in the upper left quadrant showed significantly reduced expression in naïve CD4+ T cells due to promoter hypermethylation. Genes that showed the opposite trend are shown in the bottom right quadrant. (PDF 185 kb) Additional file 21: Table S10. A list of regulatory methylation sites derived from TCGA data. (XLSX 418 kb) Additional file 22: Table S11. Pathways enriched from Gene Set Analysis of genes correlating with TCGA dataset 1 or dataset 2. (XLSX 12 kb) Additional file 23: Figure S12. Concordance of this study with *Komori* et al. (A) Each blue dot represents the methylation level of a gene. On the x-axis are the methylation differences between memory and naïve cells measured by *Komori* et al. and on the y-axis are TSS200 methylation levels measured in this study. The r^2^ value is a pearson’s correlation coefficient between the two datasets. (B) Each blue dot represents the methylation level of a gene. On the x-axis are the methylation differences between memory and naïve cells where *Komori* et al. provided quantitative values and on the y-axis are the TSS200 methylation levels measured in this study. The r^2^ value is a pearson’s correlation coefficient between the two datasets. (C) Genes identified in *Komori* et al. as displaying inverse correlation between methylation levels and transcription are plotted. On the left axis and shown in blue are the log_2_ transformed fold change of transcript levels between memory and naïve cells measured by *Komori* et al. and on the right axis and shown in green are log_2_ transformed fold change of transcript levels between memory and naïve cells measured in this study. (D) The correlation between the log_2_ transformed fold changes of genes between memory and naïve cells measured in each study is shown. Each blue dot represents a gene and the r^2^ is a pearson’s correlation coefficient. (PDF 1410 kb) Additional file 24: Figure S13. Cell surface proteins of naïve CD4+ T cells and their expression compared to resting memory CD4+ T cells (A) Cell surface proteins found in this study are compared against those found by *Graessel* et al. (B) iTRAQ measurements within this study are shown for cell surface proteins that *Graessel* et al. found to be increased in activated naïve CD4+ T cells. (PDF 75 kb) Additional file 25. Supplemental methods. (DOC 41 kb) Additional file 26: Figure S14. Purity of naïve CD4+ T cells and memory CD4+ T cells. (A) FACS-plot showing the purity of naïve CD4+ T cells. (B) FACS-plot showing the purity levels of memory CD4+ T cells. (PDF 71 kb)

## Abbreviations

FPKM: Fragments per kilobase of exon per million fragments mapped
iBAQ: Intensity based absolute quantitation
ORFs: Open reading frames
TSS: Transcription start site
TCGA: The cancer genome atlas
PBMCs: Peripheral blood mononuclear cells
CG: Complete genomics
lncRNA: NONCODE curated long non coding RNAs
SCX: Strong-cation exchange
bRPLC: Basic reversed-phase liquid chromatography
FDR: False discovery rate
