# Supplementary material for: A multi-omic analysis of human naïve CD4+ T cells
Source: BMC Syst Biol. 2015 Nov 6;9:75. doi: 10.1186/s12918-015-0225-4 (PMC4636073; doi:10.1186/s12918-015-0225-4)
Supplement: Additional file 1: Figure S1. — Data generated on naïve CD4+ T cells and memory CD4+ T cells. Basic metrics assaying the quality of generated data are provided for each dataset generated. (PDF 60 kb) [file 12918_2015_225_MOESM1_ESM.pdf]

Figure S1

DNA

Whole Genome Sequencing
- Peripheral Blood Mononuclear Cells
  - 6.78 Billion Mapped Reads
  - 34x coverage

Targeted DNA Sequencing
- Naïve CD4+ T cells
  - 1.08 million reads
  - 283x coverage per site

Methylation Array
- Illumina 450K BeadChip
  - Naïve CD4+ T cells
    - 2 technical replicates
  - Memory CD4+ T cells
    - 2 technical replicates

RNA

RNA-Seq
- Naïve CD4+ T cells
  - 67.2 million reads
  - 33 average sequence phred score
- Memory CD4+ T cells
  - 137 million reads
  - 34 average sequence phred score

Targeted RNA-Seq
- Agilent HaloPlex
  - Naïve CD4+ T cells
    - Biological replicate 1
      - 1.34 million reads
      - 316x coverage per site
    - Biological replicate 2
      - 1.96 million reads
      - 436x coverage per site

Small RNA-Seq
- Naïve CD4+ T cells
  - 13.8 million reads
  - 36 average sequence phred score

Gene Expression Array
- PrimeView HG-U133
  - Naïve CD4+ T cells
    - 3 technical replicates
  - Memory CD4+ T cells
    - 3 technical replicates

Protein

Global Proteomics
- iTRAQ
  - 365,129 tandem mass spectra
- Naïve CD4+ T cells
  - 1,702,317 tandem mass spectra

Phosphoproteomics
- Naïve CD4+ T cells
  - 311,704 tandem mass spectra
- Memory CD4+ T cells
  - 250,190 tandem mass spectra
