# Supplementary figures and images for: A multi-omic analysis of human naïve CD4+ T cells
Source: BMC Syst Biol. 2015 Nov 6;9:75. doi: 10.1186/s12918-015-0225-4 (PMC4636073; doi:10.1186/s12918-015-0225-4)

# Figure S2

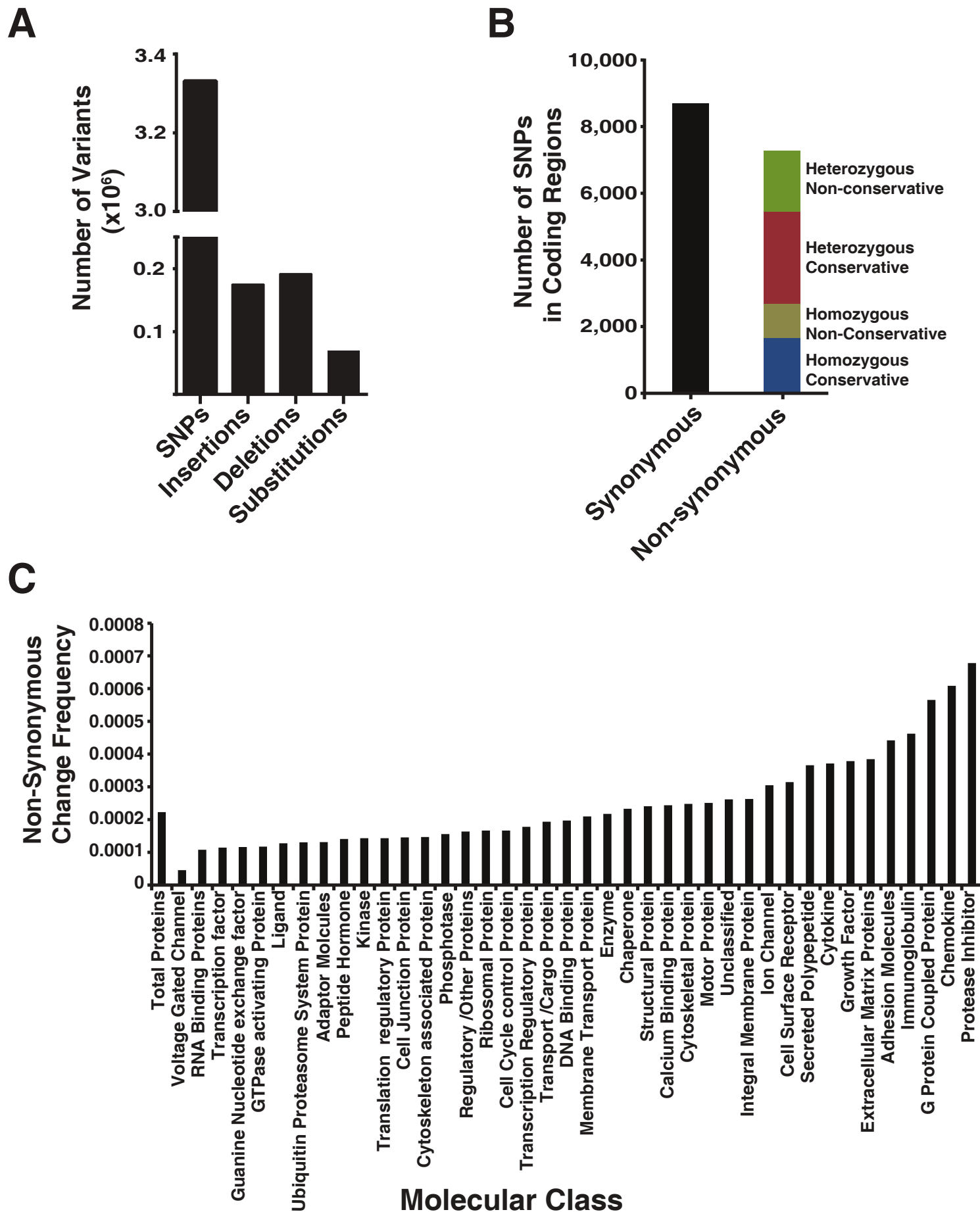

Supplement: Additional file 2: Figure S2. — Sequence variants identified in the genome and classification of SNPs in protein coding regions. (A) Total number of SNPs, insertions, deletions and substitutions identified from whole genome sequencing data. (B) Proportion of synonymous and non-synonymous SNPs identified in the coding region of the genome among non-synonymous SNPs (nsSNPs), proportion of conservative and non-conservative homozygous and heterozygous changes are shown. (C) nsSNP frequency across various molecular classes of genes. (PDF 68 kb) [file 12918_2015_225_MOESM2_ESM.pdf]

# Figure S4

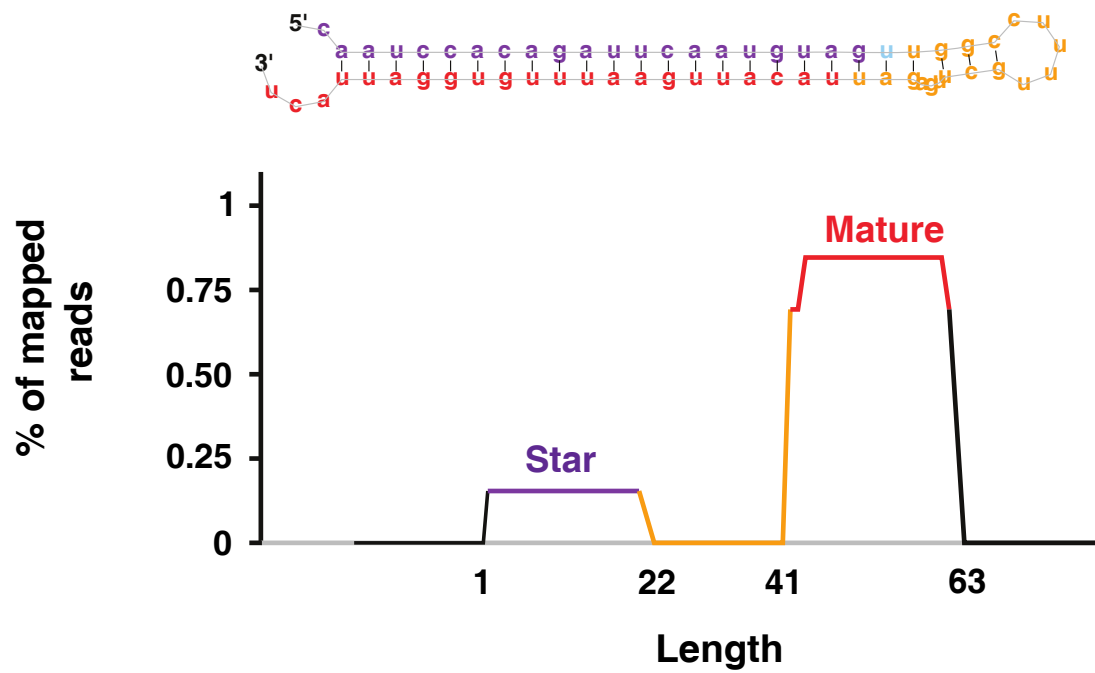

Supplement: Additional file 7: Figure S4. — Example of a miRNA identified and the distribution of reads across a predicted hairpin structure. Putative novel miRNA identified from small RNA-Seq. Predicted stem loop structure, mature region and star regions are shown. (PDF 50 kb) [file 12918_2015_225_MOESM7_ESM.pdf]

Figure S5

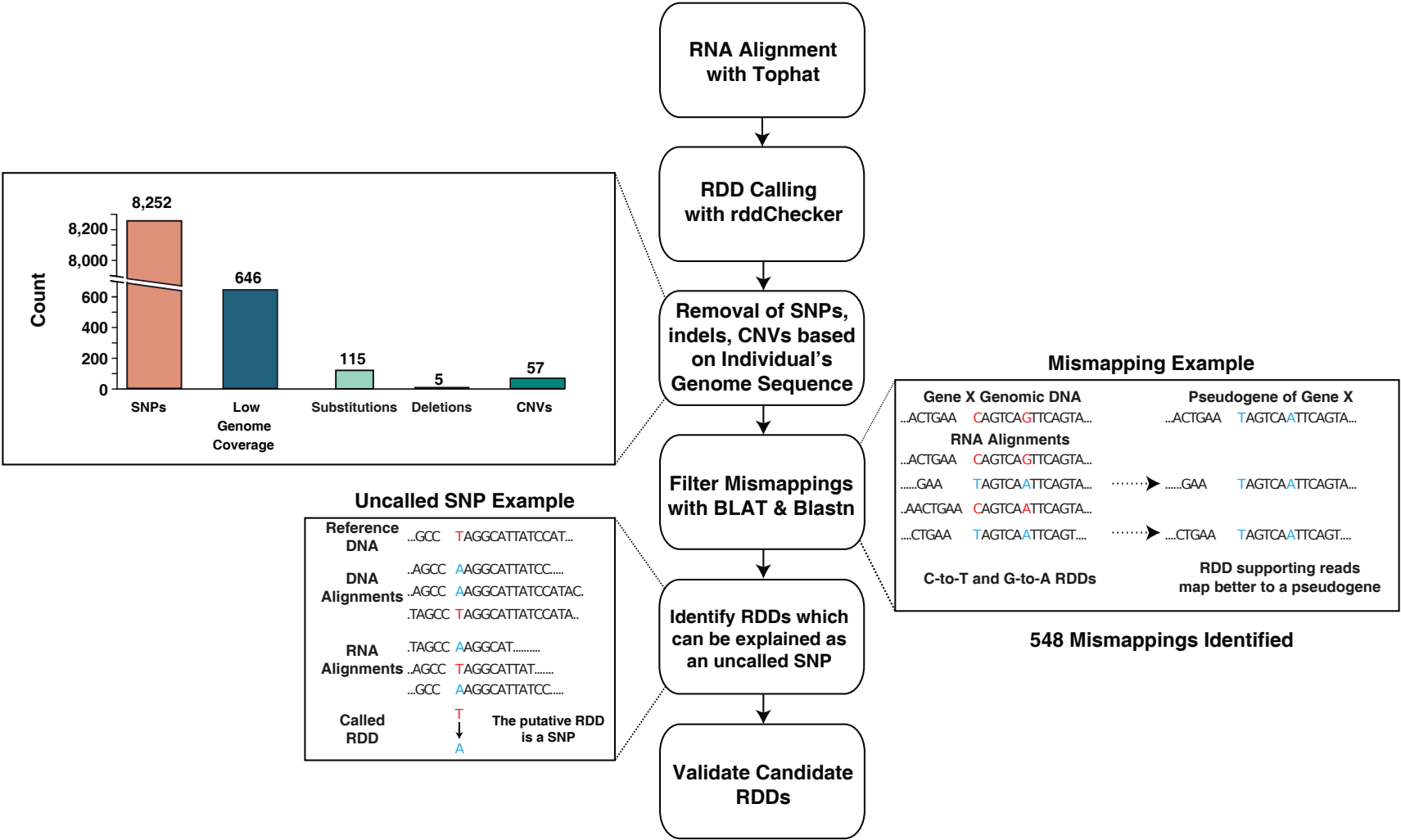

Supplement: Additional file 11: Figure S5. — Strategy employed for determining potential RNA editing sites . The workflow that was followed to filter out false positive RNA-editing sites is shown. Examples of false positives due to mismapping of reads and uncalled SNPs is demonstrated with sequence alignment. Abbreviations (SNP: single nucleotide polymorphism; RDD: RNA-DNA difference; CNV: copy number variation). (PDF 65 kb) [file 12918_2015_225_MOESM11_ESM.pdf]

# Figure S6

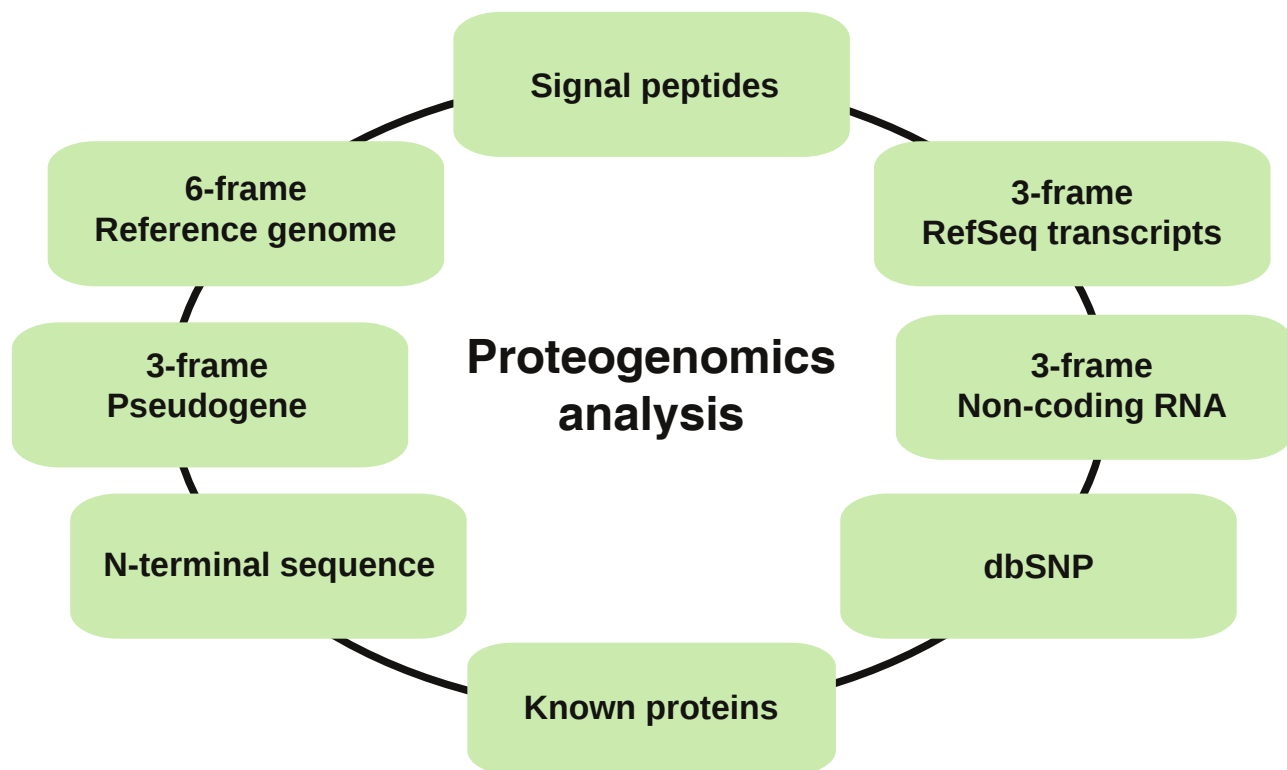

Supplement: Additional file 12: Figure S6. — Proteogenomic pipeline. Custom protein databases that were used for searching tandem mass spectrometry data for proteogenomic annotation. (PDF 59 kb) [file 12918_2015_225_MOESM12_ESM.pdf]

**Figure S7**

**Proteome and  
Transcriptome**

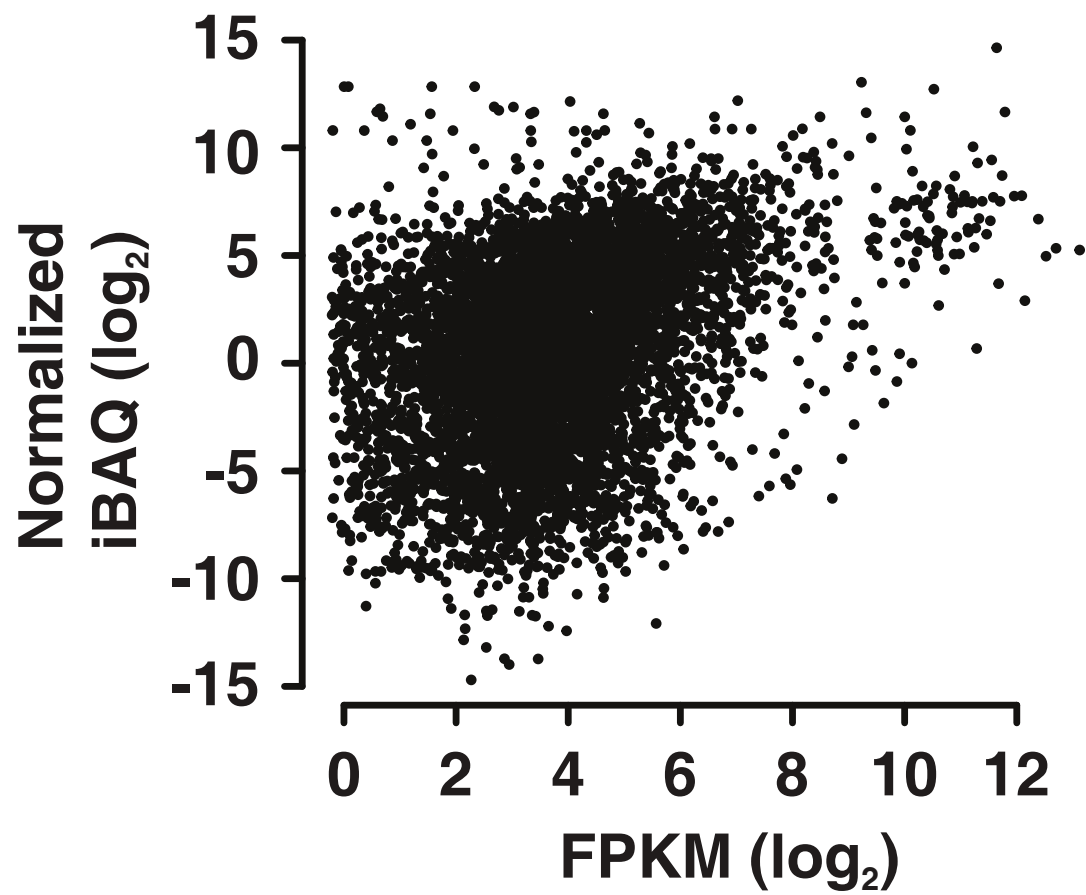

Supplement: Additional file 16: Figure S7. — Comparison of a protein’s abundance versus its transcriptional abundance. Scatterplot of protein abundance versus transcript abundance is shown. Transcript abundance is represented as FPKM (log2) on the x-axis and protein abundance is represented as normalized iBAQ (log2) on the y-axis. (PDF 487 kb) [file 12918_2015_225_MOESM16_ESM.pdf]

# Figure S8

**A**

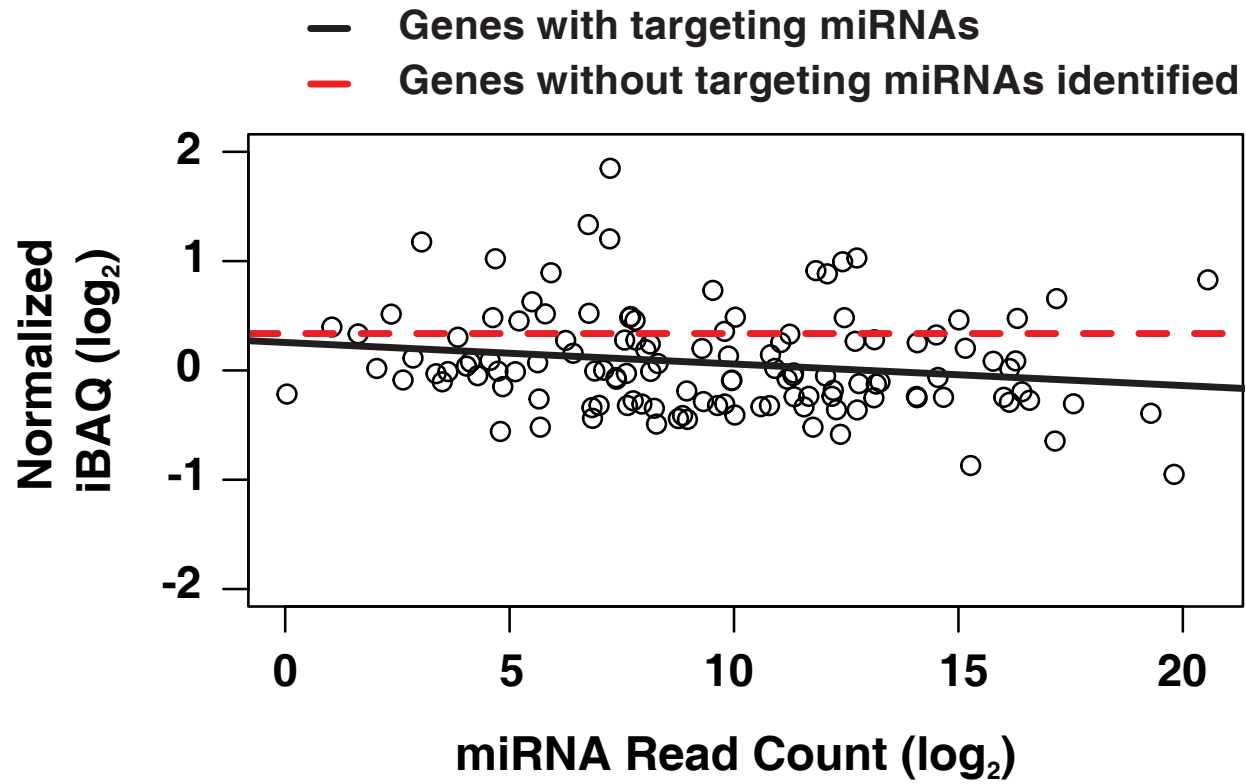

**B**

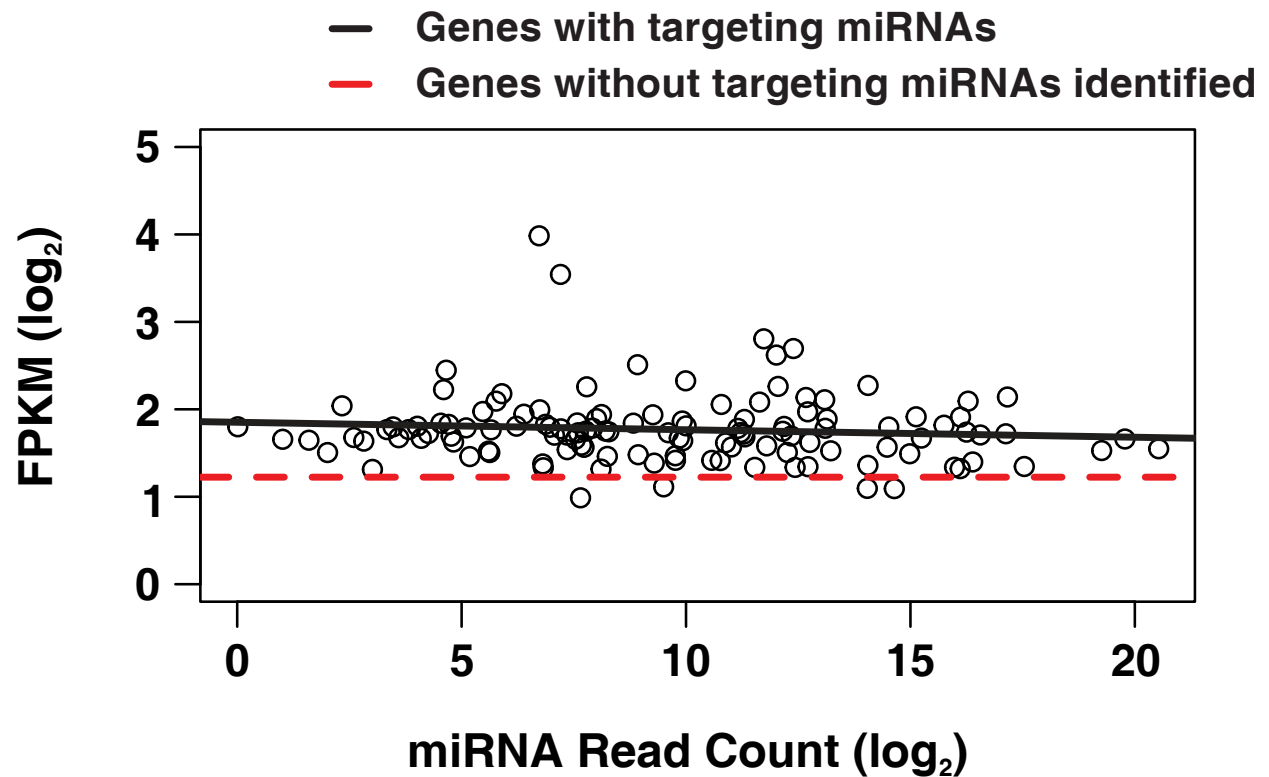

Supplement: Additional file 17: Figure S8. — Correlation between miRNA read count, transcript, and protein abundance. (A) Protein abundance versus miRNA read count. The x axis for each circle represents all miRNAs identified with a given read count while the y axis corresponds to the average iBAQ value of the genes targeted by those miRNAs with the specified read count. The dashed red line represents the iBAQ value of genes that are not targeted by any miRNAs identified in this study and serves as a background, reference level of protein expression. The black line is a linear regression of iBAQ values versus the miRNA read count. (B) Transcript abundance versus miRNA read count. The x axis for each circle represents all miRNAs identified with a given read count while the y axis corresponds to the average FPKM value of the genes targeted by those miRNAs with the specified read count. The dashed red line represents the FPKM levels of genes that are not targeted by any miRNAs identified in this study and serves as a background, reference level of transcript abundance. The black line is a linear regression of FPKM values versus miRNA read count. There is no obvious correlation between FPKM values and miRNA read counts. (PDF 150 kb) [file 12918_2015_225_MOESM17_ESM.pdf]

# Figure S9

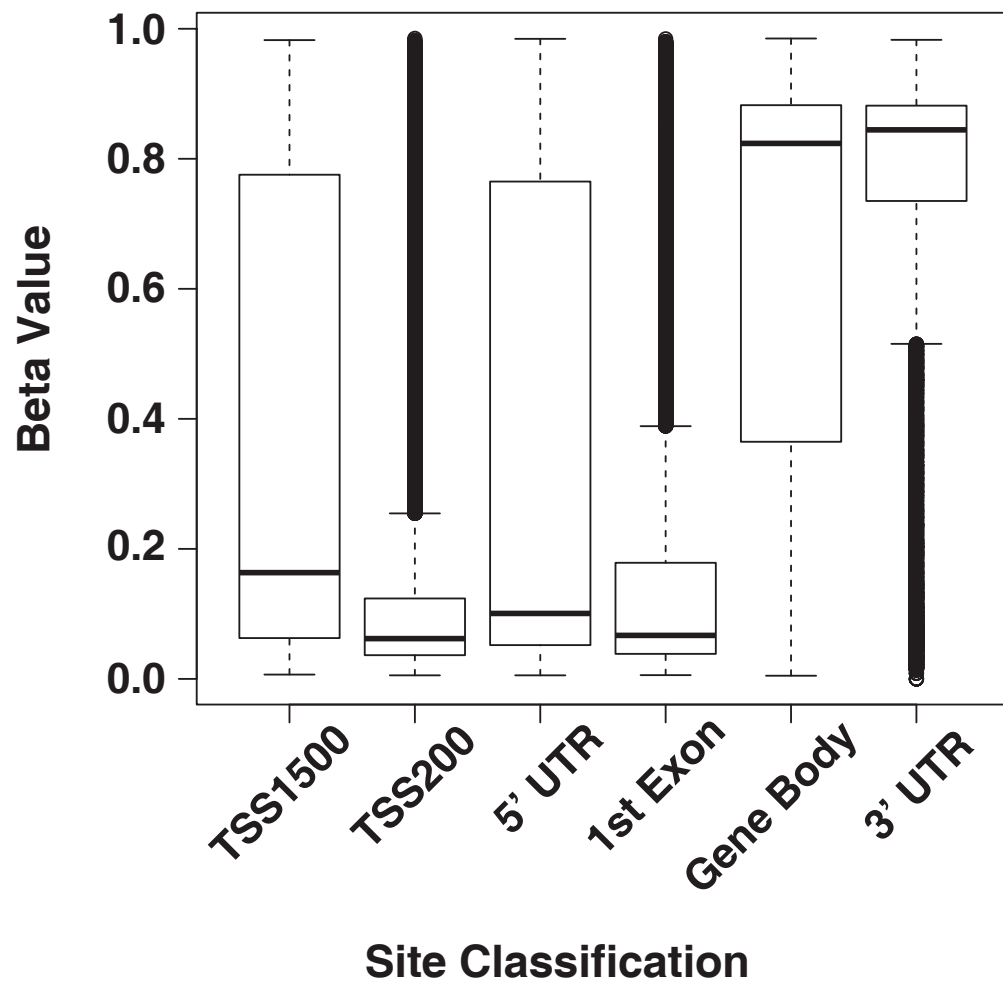

Supplement: Additional file 18: Figure S9. — DNA methylation pattern across various gene features on all protein coding genes in naïve CD4+ T cells. The x-axis represents various gene features in protein coding genes (TSS1500 - 1,500 bp upstream of transcription start site, TSS200 - 200 bp upstream of transcription start site, 5′ UTR – 5′ untranslated region, 1st exon, gene body and 3′ UTR – 3′ untranslated region) and the y-axis represents beta value which is an estimate of methylation level. Box-Whisker plots show median methylation levels at each gene feature across all protein coding genes in naïve CD4+ T cells. (PDF 1313 kb) [file 12918_2015_225_MOESM18_ESM.pdf]

# Figure S10

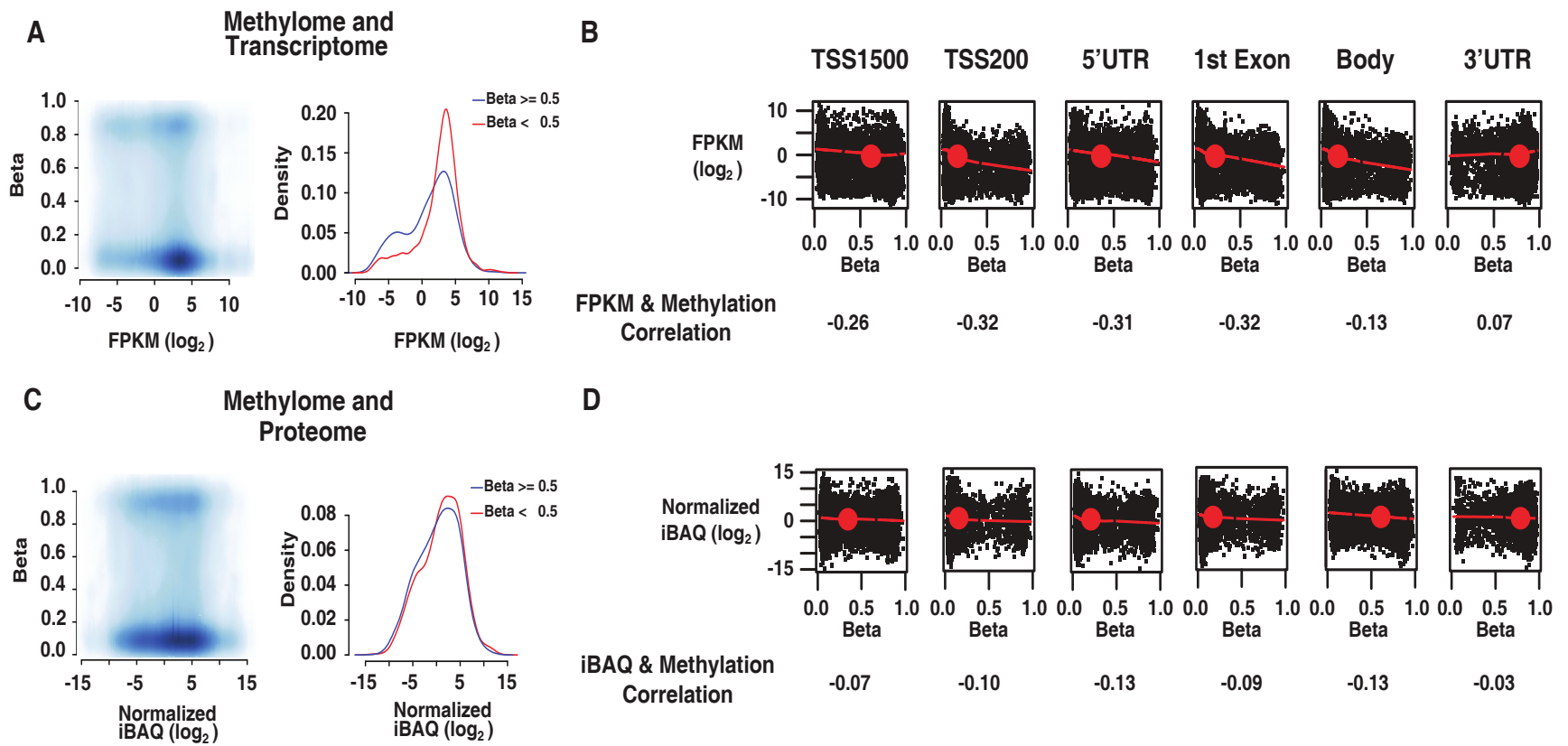

Supplement: Additional file 19: Figure S10. — Correlation of methylation level at different gene features with transcript and protein expression levels. (A) Promoter methylation level and corresponding transcript abundance Methylation levels are represented by Beta values and transcript abundance is represented by FPKM values. (B) Scatterplots showing methylation levels at different gene features and transcript abundance. Methylation levels are represented on the x-axis and transcript abundance is represented on the y-axis. The red dot represents the mean methylation levels and transcript abundances. Spearman’s correlation coefficent is represented below each scatterplot. (C) Promoter methylation level and corresponding protein abundance (iBAQ values from Fig. 4). (D) Scatterplots showing methylation levels at different gene features and protein abundance. Methylation levels are represented on the x-axis and protein abundance is represented on the y-axis. The red dot represents the mean methylation levels and protein abundances. Spearman’s correlation coefficent is represented below each scatterplot. (PDF 1087 kb) [file 12918_2015_225_MOESM19_ESM.pdf]

# Figure S11

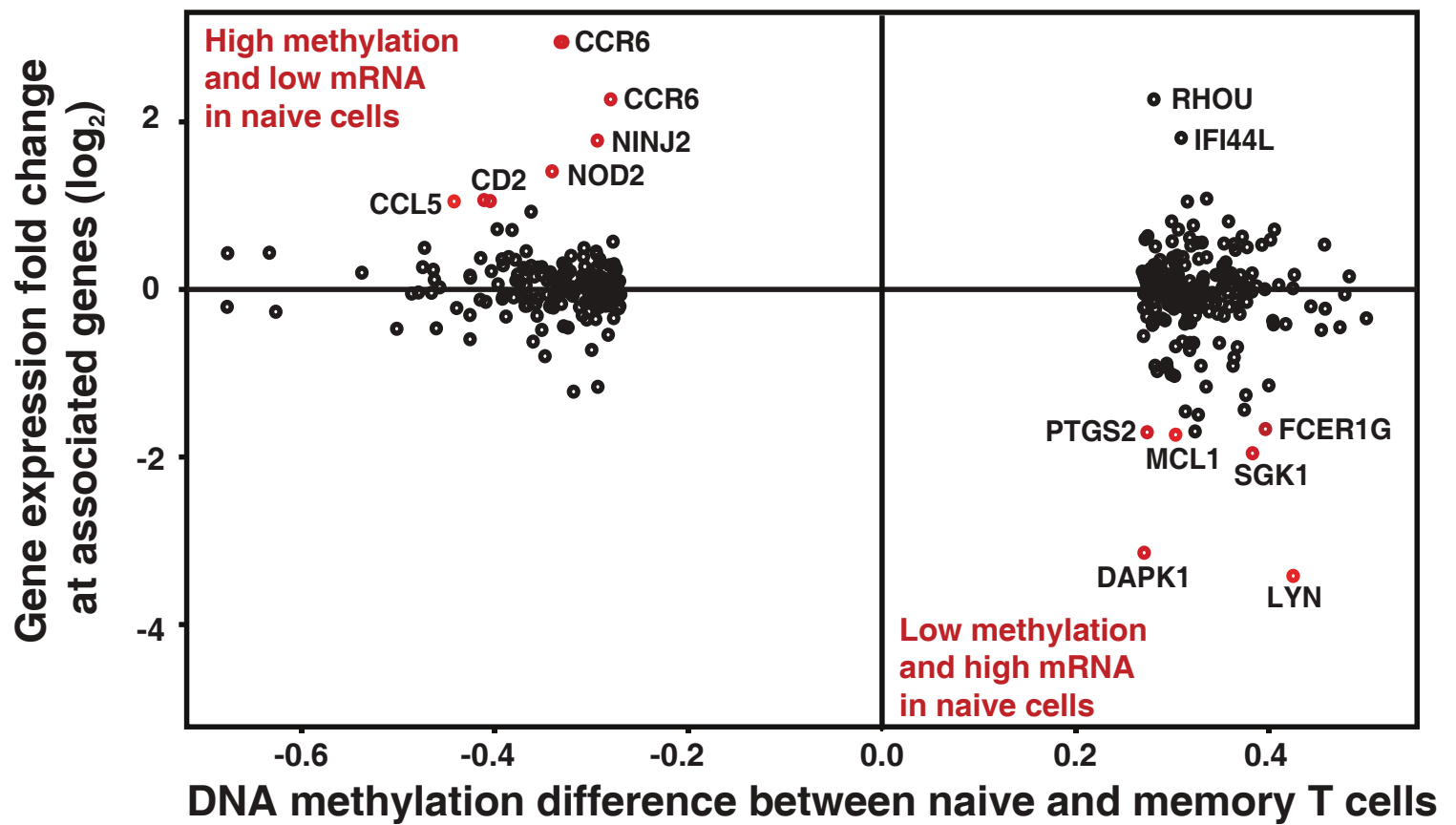

Supplement: Additional file 20: Figure S11. — Correlation of methylation and transcript levels of genes between memory and naïve CD4+ T cells. (A) Intersection of promoter methylation and mRNA expression data from naïve and memory T cells. Genes marked in red in the upper left quadrant showed significantly reduced expression in naïve CD4+ T cells due to promoter hypermethylation. Genes that showed the opposite trend are shown in the bottom right quadrant. (PDF 185 kb) [file 12918_2015_225_MOESM20_ESM.pdf]

# Figure S12

**A**

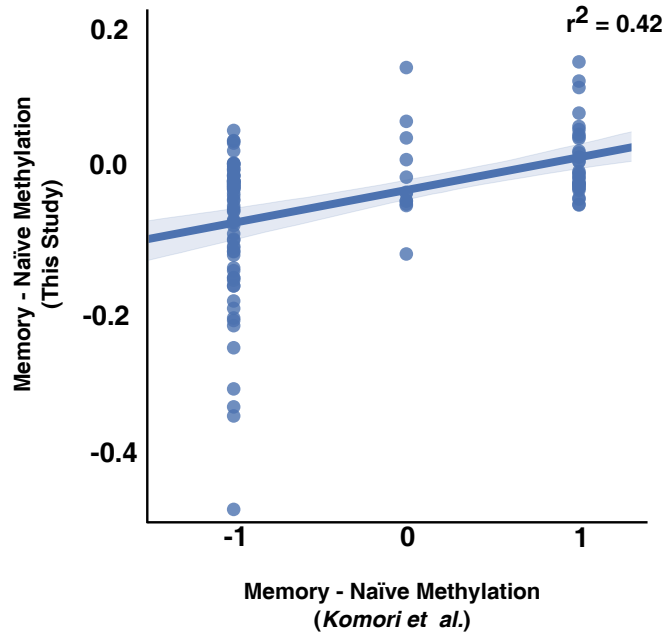

**B**

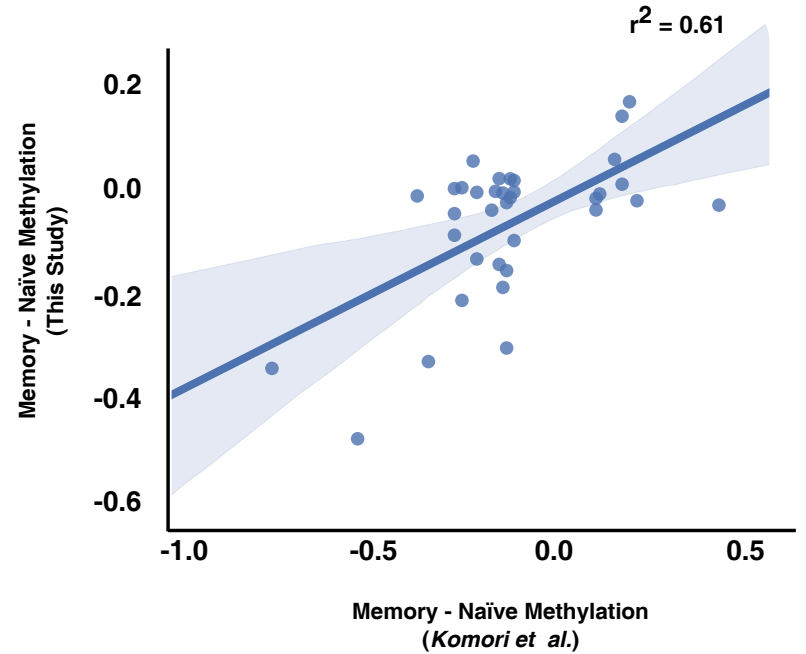

**C**

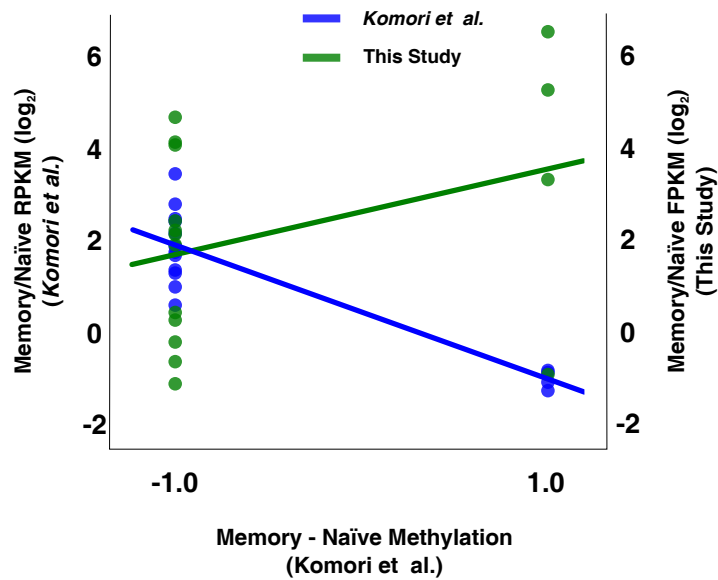

**D**

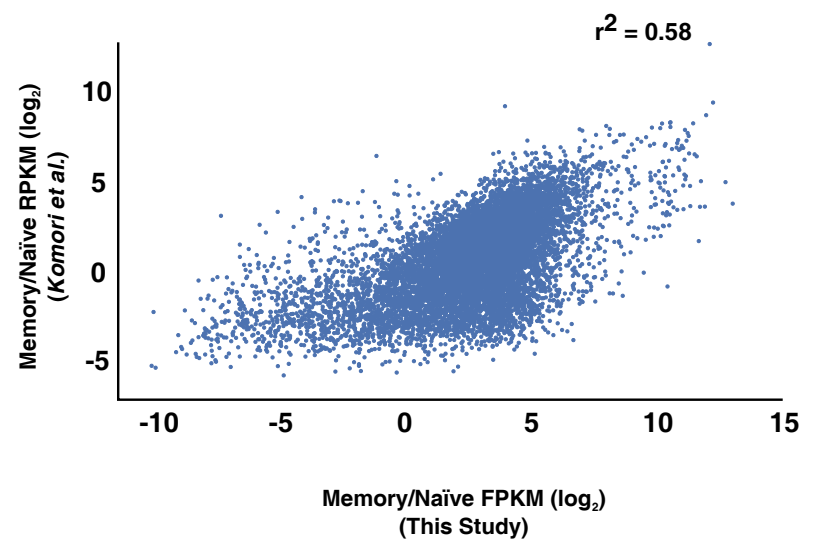

Supplement: Additional file 23: Figure S12. — Concordance of this study with Komori et al. (A) Each blue dot represents the methylation level of a gene. On the x-axis are the methylation differences between memory and naïve cells measured by Komori et al. and on the y-axis are TSS200 methylation levels measured in this study. The r2 value is a pearson’s correlation coefficient between the two datasets. (B) Each blue dot represents the methylation level of a gene. On the x-axis are the methylation differences between memory and naïve cells where Komori et al. provided quantitative values and on the y-axis are the TSS200 methylation levels measured in this study. The r2 value is a pearson’s correlation coefficient between the two datasets. (C) Genes identified in Komori et al. as displaying inverse correlation between methylation levels and transcription are plotted. On the left axis and shown in blue are the log2 transformed fold change of transcript levels between memory and naïve cells measured by Komori et al. and on the right axis and shown in green are log2 transformed fold change of transcript levels between memory and naïve cells measured in this study. (D) The correlation between the log2 transformed fold changes of genes between memory and naïve cells measured in each study is shown. Each blue dot represents a gene and the r2 is a pearson’s correlation coefficient. (PDF 1410 kb) [file 12918_2015_225_MOESM23_ESM.pdf]

# Figure S13

**A**

*Graessel et. al.*  
Surface Atlas

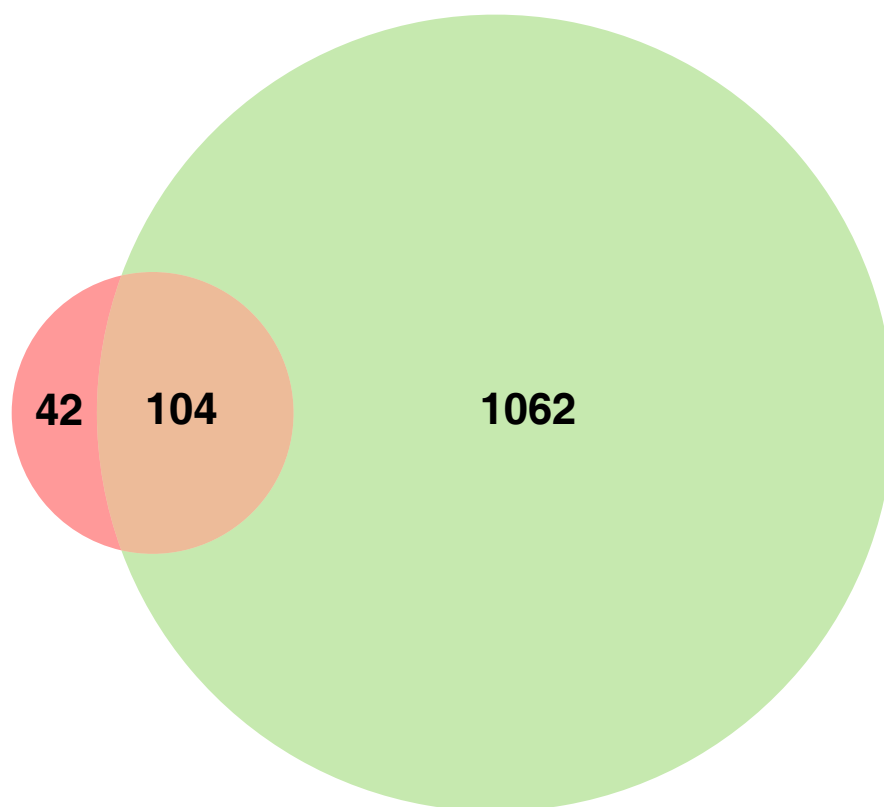

**This Study**

**B**

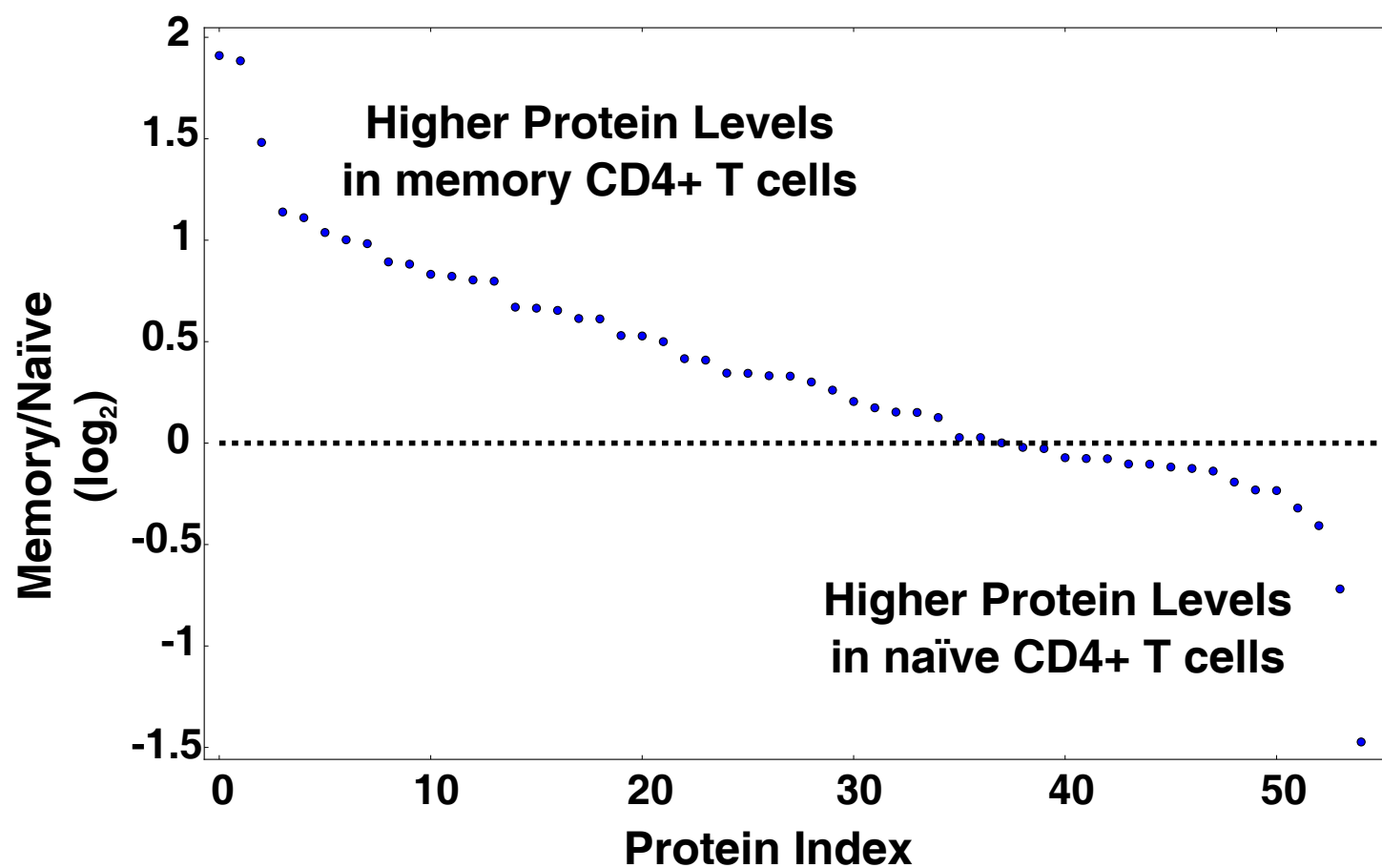

Supplement: Additional file 24: Figure S13. — Cell surface proteins of naïve CD4+ T cells and their expression compared to resting memory CD4+ T cells (A) Cell surface proteins found in this study are compared against those found by Graessel et al. (B) iTRAQ measurements within this study are shown for cell surface proteins that Graessel et al. found to be increased in activated naïve CD4+ T cells. (PDF 75 kb) [file 12918_2015_225_MOESM24_ESM.pdf]

# Figure S14

**A**

**Naïve CD4 T Cells CD4+CD25-CD45RA+**

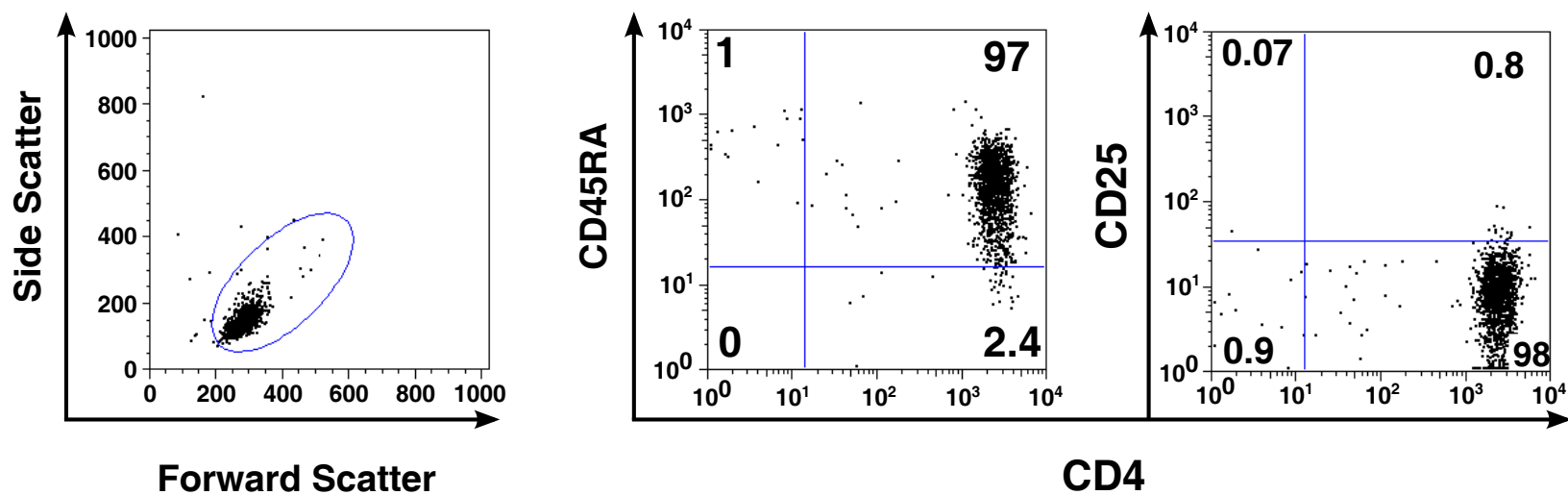

**B**

**Resting Memory CD4 T Cells CD4+CD25-CD45RA+**

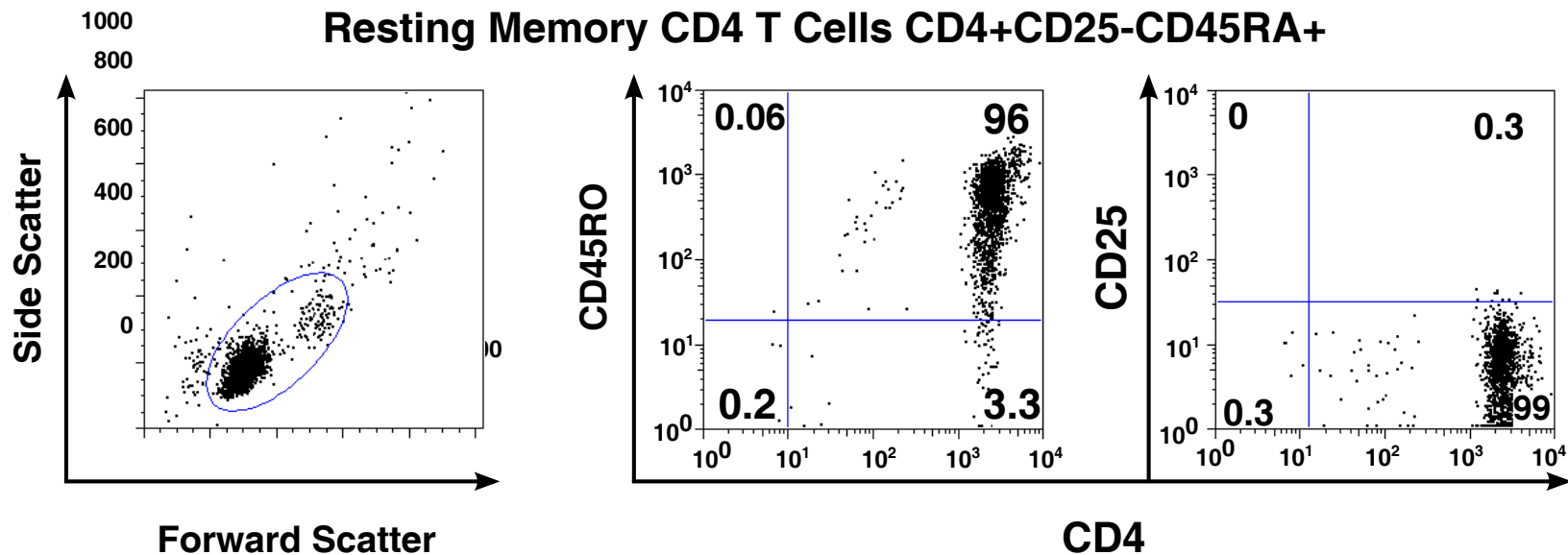

Supplement: Additional file 26: Figure S14. — Purity of naïve CD4+ T cells and memory CD4+ T cells. (A) FACS-plot showing the purity of naïve CD4+ T cells. (B) FACS-plot showing the purity levels of memory CD4+ T cells. (PDF 71 kb) [file 12918_2015_225_MOESM26_ESM.pdf]
