# Supplementary material for: A multi-omic analysis of human naïve CD4+ T cells
Source: BMC Syst Biol. 2015 Nov 6;9:75. doi: 10.1186/s12918-015-0225-4 (PMC4636073; doi:10.1186/s12918-015-0225-4)
Supplement: Additional file 3: Figure S3. — Distribution of assembled transcripts based on abundance (FPKM) and corresponding read density. The x-axis represents abundance threshold (FPKM) and y-axis represents read density. The red and green lines represent the Gaussian Mixture Model that was applied to assembled transcripts. The abundance threshold (represented by vertical dotted line) separates transcripts identified as true positives on the right (supported by more reads) from those identified as potential false positives on the left (supported by less reads). The two screenshots taken from genome browser demonstrates examples of high read density and low read density. (PDF 226 kb) [file 12918_2015_225_MOESM3_ESM.pdf]

Figure S3

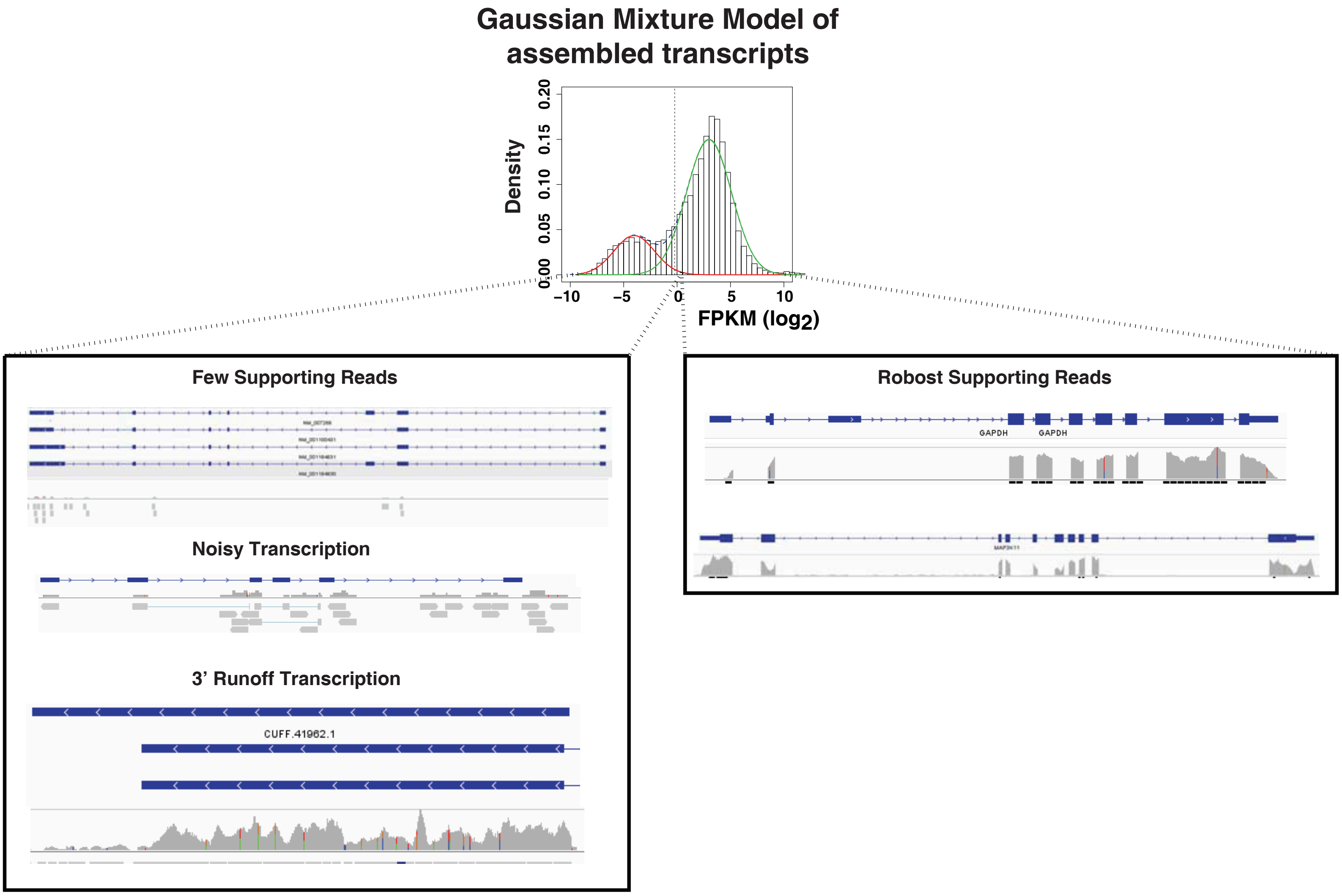
Gaussian Mixture Model of assembled transcripts
Density
0.00
0.05
0.10
0.15
0.20
−10
−5
0
5
10
FPKM (log2)
Few Supporting Reads
Noisy Transcription
3' Runoff Transcription
CUFF.41962.1
Robost Supporting Reads
GAPDH
GAPDH
