# Supplementary material for: A multi-omic analysis of human naïve CD4+ T cells
Source: BMC Syst Biol. 2015 Nov 6;9:75. doi: 10.1186/s12918-015-0225-4 (PMC4636073; doi:10.1186/s12918-015-0225-4)
Supplement: Additional file 25. — Supplemental methods. (DOC 41 kb) [file 12918_2015_225_MOESM25_ESM.doc]

**Supplemental methods**

**Cell isolation and sample preparation**

A healthy male donor was enrolled and consented for specimen donation under the institutional review board IRB protocol (IRB-J0969) through Specimen Acquisition Core (SAC) services. Peripheral blood was collected in sodium heparin containing vacutainer blood collection tubes (Becton Dickinson). Peripheral blood mononuclear cells (PBMCs) were enriched by centrifugation over Ficoll gradient at 700 g for 15 mins. Naïve CD4+ T cells were isolated from buffy coat PBMCs using magnetic beads following manufacturer’s instructions (Miltenyi Biotec #130.094.131) and the purity was assessed using FACS Calibur. Resting memory cells were isolated first by enriching pan resting CD4+ T cells followed by CD45RO depletion of resting memory CD4+ T cells. Isolated cells were washed five times with cold PBS in large volumes and stored in -80°C until use. Genomic DNA was isolated from cells using the FlexiGene DNA kit (QIAGEN) following manufacturer’s protocol. RNA was isolated from cells using RNeasy mini kit (QIAGEN) and small RNAs were isolated using miRNeasy mini Kit (QIAGEN).

**DNA sequencing and data processing**

Whole genome sequencing was performed by Complete Genomics (CG). 35 bp paired-end reads were generated by CG and mapped to human reference genome (hg19). Coverage and reference score files were used to assess coverage statistics and CG annotated structural nucleotide variants (SNV). CCDS proteins were classified based on molecular class assignment in HPRD and classes containing <50 proteins were excluded. In-house python scripts were used to map nsSNVs across various molecular classes. The following formula was used to calculate the number of nsSNVs per residue pertaining to various molecular classes: $Per residue frequency f=\frac{\left(Number of nsSNVs mapped to molecular class X genomic coordinates\right)}{\left(\left(Total number of bases covering molecular class X\right)/3\right)}$

**RNA Sequencing and analysis**

RNA Sequencing was carried out on total RNA purified from naïve CD4+ T cells following Illumina’s recommendation for RNA-Seq. RNA-Seq library was constructed using Illumina’s TruSeq RNA sample preparation kit. 500 ng total RNA was subjected to poly(A)+ selection and fragmentation. Following first and second strand synthesis, the cDNA was subjected to end repair, adenylation of 3’ ends, adapter ligation and 15 cycles of PCR amplification. One of 6 unique indices was used in each individual sample. The libraries were sequenced on HiScanSQ generating 33.6 million 100 bp paired-end reads. Reads were aligned using Tophat v. 2.0.10 and assembled into transcripts utilizing a concatenation of Illumina’s iGenomes (retrieved August 11, 2011), Mark Gerstein’s Pseudogene annotations (v. 61), and NONCODE long non coding RNA (lncRNA) (retrieved August 6, 2012) annotations [1,2]. An FPKM expression cutoff was derived by fitting a Gaussian Mixture Model and taking the mean + 2 standard deviations of the left peak as an expression threshold.

**miRNA sequencing and analysis**

Small RNAs were gel excised and an adapter sequence was ligated prior to PCR amplification. cDNA constructs were purified and sequenced on HiSeq2000 as single-end reads. 13.6 million miRNA-Seq reads were generated of which 11.5 million reads were ≥18 bp following adapter trimming. Following this, 3.8 million reads corresponding to contaminating sequences such as tRNAs, snoRNAs, and mRNAs were removed, leaving 7.6 million reads for alignment. Mature human miRNAs from miRBase 20 as well as miRNAs from other primates (chimpanzee, orangatang, and gorilla) was used as a reference in miRDeep2 to identify potential miRNAs expressed in naïve CD4+ T cells [3]. Potential targets of miRNAs expressed in CD4+ T cells were identified using miRanda, PicTar, and experimentally derived pairings found in miRTarBase [4–6]. We compared relative transcript and protein levels of predicted miRNA targets in our dataset with that of those that were not predicted as targets. This allowed us to explore predominant forms of miRNA mediated gene regulation (either at transcriptional level or translational level) in an unperturbed system.

**DNA methylation analysis**

DNA was purified from naïve CD4+ T cells or resting memory CD4+ T cells, bisulfite converted and analyzed on Illumina’s HumanMethylation 450 BeadChip Array [7]. Raw data was normalized using the methylumi package obtained through Bioconductor and run using R statistical software [8,9]. Beta values of technical replicates were normalized and averaged. Differential DNA methylation was quantified by the difference of averaged DNA methylation values between the two groups for each probe and grouped into genomic regions. RMA was used for all array gene expression analysis and difference in expression was computed using average values from replicate runs between the two groups (equivalent to average log2 ratio) [10]. Quality assessment metrics did not indicate the exclusion of any DNA methylation or gene expression arrays. Top DMRs (i.e. those exceeding area 0.27 (number of probes x average difference)) closest to genes and before the nearest TSS were plotted against transcript fold change. To identify CpG sites which are known to regulate gene expression (i.e. sites exhibiting negative correlation between expression and DNA methylation), we used the Spearman correlation coefficient to evaluate the association between DNA methylation and mRNA expression in primary tumors from the Cancer Genome Atlas (TCGA, <https://tcga-data.nci.nih.gov/tcga/>) [11]. We utilized non-embargoed TCGA samples that employed Illumina HumanDNA methylation450K platform to analyze DNA methylation and HiSeq2000 for RNA Sequencing; breast cancer with 524 samples and endometrial cancer with 225 samples (http://cancergenome.nih.gov/). From these datasets, we derived an informer set by identifying genes that showed inverse relationship between promoter methylation and gene expression. We derived DNA methylation and corresponding gene expression differences between naïve and memory CD4+ T cells at CpG sites of this gene subset.

**Nuclear preparation**

Freshly isolated naïve CD4+ T cells were used to isolate nuclei. In brief, cells were washed three times in 10 volumes of cold PBS and centrifuged at 150 g. The cell pellet was resuspended in 1 ml of hypotonic lysis buffer (20 mM HEPES, 10 mM KCl, 2 mM MgCl_2_, 2 mM CaCl_2_, pH7.4, protease inhibitor) and kept on ice for 15 min. The cells were ruptured by nitrogen cavitation method by pressurizing at 1,500 psi under nitrogen gas for 15 min on ice. Crude nuclei were collected by centrifugation at 1,000 g for 10 min at 4°C and the pellet was washed once with 1 ml nuclear wash buffer (250 mM sucrose in hypotonic buffer). Nuclei were finally collected by centrifuging at 1,000 g for 10 min at 4°C and stored at -80°C until further use.

**Protein level fractionation and trypsin digestion**

Whole cell lysate of naïve CD4+ T cells was resolved by SDS-PAGE and stained with colloidal Coomassie Blue. After removing excess stain, the lane was cut into 30 gel bands, each of which was subjected to an in-gel tryptic digestion protocol. In brief, reduction was carried out using 10 mM DTT followed by alkylation in dark using 20 mM iodoacetamide. In-gel tryptic digestion was carried out using sequencing grade trypsin (Promega) at 1:50 of enzyme to protein ratio at 37°C overnight. Peptides were extracted from the gel pieces, dried, and analyzed by LC-MS/MS. Protein fractionation was also carried out on GELFREE 8100 fractionation system according to manufacturer’s protocol. In brief, 200 µg of lysate was loaded on a 10% gel-filter and 120 µl fractions were collected following manufacturer’s time table. Each fraction was then subjected to SDS-PAGE, followed by in-gel tryptic digestion as described above.

**Peptide level fractionation using SCX or bRPLC**

Peptide level fractionation was carried by strong-cation exchange (SCX) as well as basic reversed-phase liquid chromatography (bRPLC) following in-solution trypsin digestion of protein lysate. Proteins were reduced using 5 mM DTT, alkylated using 10 mM iodoacetamide and digested with trypsin (Promega) at 37°C overnight (enzyme:substrate ratio of 1:20). Peptides were desalted using Sep-Pak C18 cartridge (Waters) and lyophilized. Lyophilized peptides were resuspended in SCX solvent A and loaded on polysulfoethyl A strong cation exchange column (PolyLC; 200×2.1 5um, 200Å). A total of 96 fractions were collected into 96-well plate and concatenated into two fractions (early fraction and later fraction), each of which was vacuum-dried and fractionated again by bRPLC method. Dried peptides were resuspended using bRP solvent A and loaded onto a bRPLC column (X-Bridge, Waters) at high pH. Each of these samples were separated into 96 fractions. Ninety six fractions from peptides that eluted early in SCX were concatenated into 14 fractions while those that eluted late were concatenated into 18 fractions. The samples were vacuum dried and reconstituted in appropriate buffer for LC-MS/MS analysis and iTRAQ based quantitative proteomics experiments.

**LC-MS/MS analysis**

LC-MS/MS analysis was performed using an LTQ-Orbitrap Elite mass spectrometer (Thermo Fisher Scientific) interfaced with EASY-nLC (Thermo Fisher Scientific) or an LTQ-Orbitrap Velos interfaced with Agilent 1100 series HPLC system. Peptides were loaded onto a trap column (2 cm×75 µm ID) packed in-house with C18 material (5 µm Magic C18 AQ, Michrom) and separated on an analytical column (20 cm×75 µm ID) packed in-house with the same material. For HPLC, 0.1% formic acid in H_2_O was used as solvent A and 0.1% formic acid in acetonitrile was used as solvent B. Peptides were eluted into nano-electrospray ionization source using pico-tip emitter 10 µm tip (New Objective; Woburn, MA; 360 µm OD×20 µm) at 2 kV. Precursor ions and fragment ions were measured in *high-high* mode, with resolution settings of 120,000 and 30,000 for the LTQ-Orbitrap Elite and resolution settings of 60,000 and 15,000 for the LTQ-Orbitrap Velos, respectively, in a data-dependent manner. Only multiply charged precursor ions were selected for fragmentation. High energy collision induced dissociation (HCD) method was employedfor peptide fragmentation by using normalized collision energy of 32% on LTQ-Orbitrap Elite and 35% or 40% on LTQ-Orbitrap Velos. For iTRAQ based quantitative proteomics experiments, 75 µg of in-solution digested peptides from both naïve and memory T cells were used. iTRAQ labelling was carried out using manufacturer’s protocol. Excess reagents were removed by SCX, and fractionated by bRPLC prior to LC-MS/MS analysis.

**MS Data Processing and Database searching**

Tandem MS data were processed using Proteome Discoverer 1.3 (Thermo Fisher Scientific) and searched using Mascot (version 2.2) and Sequest database search algorithms against a human RefSeq protein sequence database containing common contaminant proteins with the following search parameters: trypsin as a proteolytic enzyme with up to two missed cleavages allowed; peptide mass error tolerance of 20 ppm; fragment mass error tolerance of 0.05 Da; carbamidomethylation at cysteine as fixed modification; oxidation at methionine, protein N-termini acetylation, and deamidation of glutamine and asparagine as variable modifications; peptide identifications were filtered by setting a threshold of <1% false discovery rates using target-decoy method [12].

**Phosphopeptide enrichment and label-free quantitation**

Lysates from naïve and memory CD4+ T cells were digested using in-solution trypsin digestion protocol as described above. Phosphopeptides were enriched using TiO_2_-based enrichment protocol. Enriched phosphopeptides were desalted by Stage-Tip and subjected to LC-MS/MS using Triple-TOF mass spectrometer with a long gradient. Tandem mass spectra were searched against human protein database using Mascot and ProteinPilot and peptides were identified by setting a threshold of <1% false discovery rate. Phosphopeptide-spectrum matches were confirmed manually for each phosphopeptide identified and ambiguity of phosphosites was assessed. Phosphopeptides from naïve and memory cells were quantitated by label-free method using MassNavigator. AreaScore(L) was used to assess the confidence of the area quantitation and peptides with an AreaScore greater than 0.8 were used for comparative analysis after normalization. All area values measured in phosphoproteomics analysis were normalized based on a total summed intensity of proteomic analysis.

**Proteogenomic analysis**

Proteogenomics analysis was conducted as described previously [13,14]. In brief, we first generated several custom protein/peptide sequence databases; 6-frame translated protein sequence of the individual’s genome; 3-frame translated protein sequences of all cufflinks transcripts including psudeogenes, ncRNAs and novel transcripts; peptide sequences containing peptide-level structural variants; and peptide sequences with initiator methionine of cufflinks transcripts. Unmatched tandem mass spectra were searched in parallel against custom protein/peptide sequence databases and the peptide identifications were filtered based on 1% false discovery rate threshold using target-decoy method.

**References**

1. Karro, J. E. *et al.* (2007) Pseudogene.org: a comprehensive database and comparison platform for pseudogene annotation. *Nucleic Acids Res.* **35,** D55–D60.

2. Bu, D. *et al.* (2011) NONCODE v3.0: integrative annotation of long noncoding RNAs. *Nucleic Acids Res.* gkr1175. doi:10.1093/nar/gkr1175

3. Friedländer, M. R. *et al.* (2012) miRDeep2 accurately identifies known and hundreds of novel microRNA genes in seven animal clades. *Nucleic Acids Res.* **40,** 37–52.

4. John, B. *et al.*(2004) Human MicroRNA Targets. *PLoS Biol.* **2**.

5. Krek, A. *et al.* (2005) Combinatorial microRNA target predictions. *Nat. Genet.* **37,** 495–500.

6. Hsu, S.-D. *et al.* (2011) miRTarBase: a database curates experimentally validated microRNA–target interactions. *Nucleic Acids Res.* **39,** D163–D169.

7. Bibikova, M. *et al.* (2011) High density DNA methylation array with single CpG site resolution. *Genomics* **98,** 288–295.

8. Gentleman, R. C. *et al.* (2004) Bioconductor: open software development for computational biology and bioinformatics. *Genome Biol.* **5,** R80.

9. Davis, S. *et al.* (2012) methylumi: Handle Illumina methylation data. *R Package Version 240*.

10. Irizarry, R. A. *et al.* (2003) Exploration, normalization, and summaries of high density oligonucleotide array probe level data. *Biostat* **4,** 249–264.

11. Network, T. C. G. A. (2012) Comprehensive molecular portraits of human breast tumours. *Nature* **490,** 61–70.

12. Elias, J. E. *et al.* (2005) Comparative evaluation of mass spectrometry platforms uised in large-scale proteomics investigations. *Nat. Methods* **2,** 667–675.

13. Chaerkady, R. *et al.* (2011) A proteogenomic analysis of Anopheles gambiae using high-resolution Fourier transform mass spectrometry. *Genome Res.* **21,** 1872–1881.

14. Kelkar, D. S. *et al.* (2011) Proteogenomic Analysis of Mycobacterium tuberculosis By High Resolution Mass Spectrometry. *Mol. Cell. Proteomics MCP* **10**.
